# Tumor induces muscle wasting in mice through releasing extracellular Hsp70 and Hsp90

**DOI:** 10.1038/s41467-017-00726-x

**Published:** 2017-09-19

**Authors:** Guohua Zhang, Zhelong Liu, Hui Ding, Yong Zhou, Hoang Anh Doan, Ka Wai Thomas Sin, Zhiren J. Zhu, Rene Flores, Yefei Wen, Xing Gong, Qingyun Liu, Yi-Ping Li

**Affiliations:** 1 Department of Integrative Biology and Pharmacology, University of Texas Health Science Center at Houston (UTHealth), Houston, Texas 77030 USA; 2 Division of Endocrinology, Tongji Hospital, Tongji Medical College, Huazhong University of Science and Technology, Wuhan, 430030 China; 3 Department of Respiratory Medicine, Yixing Hospital affiliated to Jiangsu University, Yixing, 214200 China; 4 Academic and Research Affairs, University of Texas Health Science Center at Houston (UTHealth), Houston, Texas 77030 USA; 5 The Brown Foundation Institution of Molecular Medicine, University of Texas Health Science Center at Houston (UTHealth), Houston, Texas 77030 USA

## Abstract

Cachexia, characterized by muscle wasting, is a major contributor to cancer-related mortality. However, the key cachexins that mediate cancer-induced muscle wasting remain elusive. Here, we show that tumor-released extracellular Hsp70 and Hsp90 are responsible for tumor’s capacity to induce muscle wasting. We detected high-level constitutive release of Hsp70 and Hsp90 associated with extracellular vesicles (EVs) from diverse cachexia-inducing tumor cells, resulting in elevated serum levels in mice. Neutralizing extracellular Hsp70/90 or silencing Hsp70/90 expression in tumor cells abrogates tumor-induced muscle catabolism and wasting in cultured myotubes and in mice. Conversely, administration of recombinant Hsp70 and Hsp90 recapitulates the catabolic effects of tumor. In addition, tumor-released Hsp70/90-expressing EVs are necessary and sufficient for tumor-induced muscle wasting. Further, Hsp70 and Hsp90 induce muscle catabolism by activating TLR4, and are responsible for elevation of circulating cytokines. These findings identify tumor-released circulating Hsp70 and Hsp90 as key cachexins causing muscle wasting in mice.

## Abstract (web summary)

Cachexia affects many cancer patients causing weight loss and increasing mortality. Here, the authors identify extracellular Hsp70 and Hsp90, either in soluble form or secreted as part of exosomes from tumor cells, to be responsible for tumor induction of cachexia.

## Introduction

Cachexia, a wasting disease characterized by loss of skeletal muscle mass, is a complex metabolic syndrome seen in more than 50% of cancer patients. Prominent clinical features of cachexia are weight loss, inflammation, insulin resistance and increased muscle protein breakdown^1, 2^. Not only does cachexia increases patients’ morbidity and mortality through systemic wasting but it also decreases the efficacy while increasing the toxicity of chemotherapy^3^. However, there has been no standardized assessment or established treatment for cancer cachexia due to the poor understanding of its etiology. The key event that initiates muscle wasting in cancer hosts is undetermined.

The mechanism of cancer-induced loss of the host’s muscle mass is highly complex. Cachexia-inducing cancers provoke a catabolic response in muscle characterized by activation of multiple protein-degradation pathways, including the ubiquitin proteasome pathway (UPP) that degrades myofibrillar as well as specific regulatory proteins involved in muscle protein expression, and the autophagy-lysosome pathway (ALP) that degrades mitochondria and other cellular components^2^. Although activation of these protein-degradation pathways also takes place in muscle atrophy induced by fasting, disuse and denervation, cancer-induced muscle wasting displays some distinctive features including the presence of a severe systemic inflammation. Studies in animal models of cancer cachexia revealed that the p38β MAPK-C/EBPβ signaling pathway plays a central role in the activation of muscle catabolism in animal models of cancer cachexia^4, 5^, whereas the Akt-FoxO1/3 signaling pathway that regulates proteolysis in response to fasting, disuse and denervation is non-essential due to the activation of Akt^4, 6^. Akt activation was also observed in cachectic muscle of cancer patients^7, 8^. Thus, Akt does not appear to be responsible for the muscle catabolism induced by cancer. In addition, C/EBPβ-regulated E3 ubiquitin ligases atrogin1 (MAFbx) and UBR2 (E3α-II), rather than FoxO1/3-regulated E3 MuRF1, are consistently upregulated in cachectic muscle of tumor-bearing mice^4, 9, 10^. These data suggest that systemic inflammation that activates the p38β MAPK-C/EBPβ signaling pathway is critical to the activation of muscle catabolism during cancer cachexia. Nevertheless, the precise mechanism through which the catabolic pathways in muscle are remotely activated by cancer in discrete locations is still undefined.

The extramuscular mechanism through which cancer activates muscle catabolism is currently thought to be multifactorial, involving cancer-generated factors as well as host-generated factors. Many clinical trials for intervening in cancer cachexia have been conducted using diverse strategies with unsatisfactory outcomes^11^, highlighting the dire need to find the missing root cause of cancer cachexia. All cancers do not promote cachexia, but patients with particular solid tumors including lung, pancreatic, colorectal or gastric cancer are most likely to experience significant loss of skeletal muscle mass^12, 13^, suggesting that specific cancer cell-generated humoral factors play a key role in the development of cachexia as ‘cachexins’. Despite the implication of a number of humoral factors found in the cancer milieu that stimulate muscle catabolism including pro-inflammatory cytokines such as TNFα, IL-6 and IL-1β, as well as agonists of type IIB activin receptor (ActRIIB) activins^2^, to name a few, key cancer-generated cachexins that trigger muscle catabolism remain elusive.

We previously observed that conditioned medium of Lewis lung carcinoma (LLC) cells, a potent cachexia inducer, activates a catabolic response in cultured myotubes that recapitulates the muscle catabolism in LLC tumor-bearing mice^4, 10^, suggesting that LLC cells release cachexins that directly activate muscle catabolism independent of host response. Herein, we report that in screening for the catabolic components of LLC cell-conditioned medium (LCM) we found surprisingly that the catabolic activity was associated with high levels of Hsp70 and Hsp90. Furthermore, we observed elevated release of Hsp70 and Hsp90 into culture media by other prominent cachexia-inducing tumor cells of mouse or human origin, as well as in the serum of two complementary models of cancer cachexia in mice, suggesting a potential role for tumor-generated circulating Hsp70 and Hsp90 in muscle wasting. In addition, we found that cachexia-inducing tumor cells release high levels of extracellular vesicles (EVs) serving as carrier of tumor-released Hsp70 and Hsp90. Given that a number of tumor cells express high levels of cell surface Hsp70/90^14–17^ and release EVs expressing surface Hsp70/90^18–21^ that cause systemic inflammation as danger-associated molecular patterns (DAMPs)^22^, we tested the hypothesis that tumor cells induce muscle wasting by releasing high levels of extracellular Hsp70 and Hsp90. We demonstrate here that tumor cell-released EV-associated Hsp70 and Hsp90 are necessary and sufficient to induce muscle wasting. In addition, we demonstrate that tumor-released Hsp70 and Hsp90 induce muscle wasting by activating TLR4 on muscle cells. Further, we show that release of Hsp70 and Hsp90 by tumor is required for the elevation of circulating inflammatory cytokines in tumor-bearing mice. Therefore, tumor-released extracellular Hsp70 and Hsp90 are key cachexins that mediate muscle wasting, and promising therapeutic targets for defeating cancer cachexia.

## Results

### Cachectic tumors release Hsp70/90 via extracellular vesicles

To identify LLC cell-released soluble factors that induce muscle catabolism, we fractionated LCM utilizing chromatography (mono-Q anion-exchange followed by gel filtration) and screened the eluate fractions for catabolic activity on C2C12 myotubes. Surprisingly, the major catabolic activity was located in fractions with molecular weight ranging from ~70 to 100 kDa, much larger than the size of previously reported humoral factors implicated in cancer cachexia including pro-inflammatory cytokines and activins, which were all under 30 kDa. Mass spectrometry analysis revealed that the active fractions were highly enriched with Hsp70, Hsp90α and Hsp90β.

**Fig. 1:**
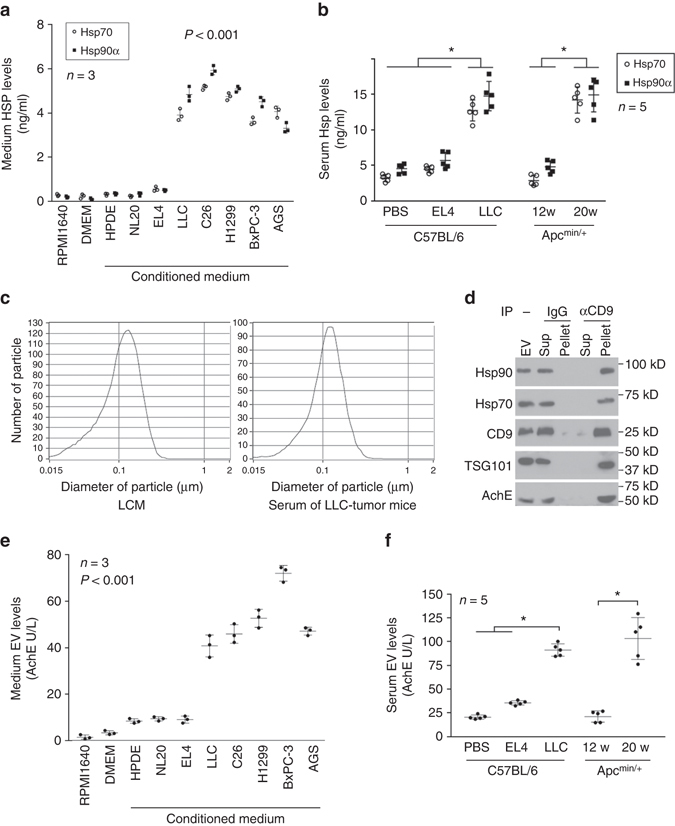
Cancer cachexia co-exists with high circulating levels of Hsp70 and Hsp90 associated with tumor cell-released EVs. **a** Cachexia-inducing tumor cells constitutively release high levels of Hsp70 and Hsp90. Hsp70 and Hsp90 content in conditioned media of cachexia-inducing tumor cells (LLC, C26, H1299, BxPC3 and AGS) are compared with that of non-cachexia-inducing tumor cell EL4 and non-tumorigenic cells (NL20, HPDC and C2C12 myotubes). All cells were incubated for 48 h in regular media (RPMI 160 or DMEM supplemented with 10% fetal bovine serum) and then analyzed for Hsp70 and Hsp90 content by ELISA. Data (*n* = 3) were analyzed by analysis of variance (ANOVA). **b** Cachexia co-exists with elevated circulating Hsp70 and Hsp90 in tumor-bearing mice. Hsp70 and Hsp90 levels in the sera of PBS-injected control, EL4 tumor- or LLC tumor-bearing C57BL/6 mice on day 21 of tumor implant, and Apc^min/+^ mice that were pre-cachectic (12 weeks of age) or cachectic (20 weeks of age) were determined by ELISA. Data (*n* = 5) were analyzed by ANOVA (for C57BL/6 mice) or Student *t*-test (for Apc^min/+^ mice). * denotes a difference among bracketed C57BL/6 mice or with pre-cachectic Apc^min/+^ mice (*P* < 0.05). **c** Particle size of EVs isolated from LCM and the serum of cachectic LLC tumor-bearing mice were analyzed using ZetaView® Nanoparticle Tracking Analyzer. **d** Tumor-released Hsp70 and Hsp90 are associated with CD9/TSG101/AchE-positive EVs. EVs were isolated from LCM and subjected to immunoprecipitation using CD9 antibody with pre-immune IgG as control. EVs, precipitated pellet and supernatant were analyzed with western blotting. **e** Cachexia-inducing tumor cells constitutively release high levels of EVs. EVs were isolated from diverse tumor cell-conditioned media and quantified by AchE activity. Data (*n* = 3) were analyzed by ANOVA. **f** Cachexia is associated with elevated circulating EVs in tumor-bearing mice. EV levels in serum of indicated mice were determined. Data (*n* = 5) were analyzed by ANOVA (for C57BL/6 mice) or Student *t*-test (for Apc^min/+^ mice). * denotes a difference among bracketed C57BL/6 mice or pre-cachectic Apc^min/+^ mice (*P* < 0.05)

To investigate whether constitutively releasing Hsp70 and Hsp90 is a common feature shared by diverse types of cancer cells that induce cachexia, we determined the levels of Hsp70 and Hsp90 in conditioned media of cancer cells known to induce cachexia including LLC (mouse lung carcinoma), C26 (mouse colon adenocarcinoma), H1299 (human non-small cell lung carcinoma), BxPC3 (human pancreatic adenocarcinoma) and AGS (human gastric adenocarcinoma) and compared them to conditioned media of mouse lymphoma cell EL4 that does not induce cachexia, as well as non-tumorigenic cells including NL20 (human lung endothelial cells) and HPDC (human pancreatic duct epithelial cells). Cell numbers at the time of medium collection are shown in Supplementary Fig. 1. Utilizing enzyme-linked immunosorbent assay (ELISA), we detected very low levels of Hsp70 and Hsp90α in conditioned media of the non-tumorigenic cells as well as EL4 lymphoma cells lacking the capacity to induce cachexia, whereas the content of Hsp70 and Hsp90α in conditioned media of the cachexia-inducing tumor cells were dramatically elevated by averaging ~10-fold (Fig. 1a). This result indicates that constitutively release of high levels of Hsp70 and Hsp90 is a common feature associated with diverse types of cachexia-inducing tumor cells of both mouse and human origin.

To evaluate whether elevated circulating Hsp70 and Hsp90 co-exist with cancer cachexia in vivo, we analyzed serum Hsp70 and Hsp90α levels in a syngeneic (LLC)^4^ and a genetic (Apc^min/+^)^9^ mouse cancer cachexia model, as well as in mice bearing the non-cachexia-inducing EL4 tumor (Supplementary Fig. 2C), all of which were in the C57BL/6 mouse background. We found that mice that developed cachexia acutely (3 weeks) due to LLC tumor xenograft had ~3–4-fold higher serum levels of Hsp70 and Hsp90α than non-tumor-bearing control mice (Fig. 1b). On the other hand, mice bearing non-cachexia-inducing EL4 tumor did not exhibit a significant elevation of serum Hsp70 and Hsp90α levels when the tumor grew to the similar size as LLC tumor in 3 weeks (Fig. 1b). Further, serum levels of Hsp70 and Hsp90α in Apc^min/+^ mice that developed cachexia chronically due to intestinal adenoma caused by the Apc^min/+^ mutation at 20-week of age^9^ were elevated ~3–4-fold but not at the pre-cachectic stage of 12-week of age (Fig. 1b). The similar increase in serum Hsp70 and Hsp90 in the two different cancer cachexia models reveals that diverse types of cachexia-inducing tumor cells release high levels of Hsp70 and Hsp90, and cancer cachexia co-exists with elevated circulating Hsp70 and Hsp90, suggesting a potential role for the two HSPs in cancer cachexia.

HSP molecules do not possess the signal peptide sequence that mediates protein secretion via the conventional route from the endoplasmic reticulum to the Golgi and release from the cell^23^. Hsp70 and Hsp90 are released from various cells via unconventional mechanisms, one of which involves EVs that express HSPs on their surface, which can activate plasma membrane HSP receptors on recipient cells^18–21^. Hence, we investigated whether the detected extracellular Hsp70 and Hsp90 were associated with tumor cell-released EVs. We analyzed EVs isolated from LCM and serum of LLC tumor-bearing mice. Utilizing electron microscopy (EM) and ZetaView® Nanoparticle Tracking Analyzer we observed that the EV preparations contained primarily double-layered vesicles (Supplementary Fig. 3A) with a median diameter of ~110 nm (Fig. 1c). Hsp70 and Hsp90 were detected in isolated EVs that expressed CD9, TSG101 and acetylcholinesterase (AchE), which are proteins often found in EVs^24^ (Fig. 1d). Because all existing common isolation methods for EVs do not achieve complete purity^24^, isolated EVs were further examined by immunoprecipitation using an antibody against CD9. Hsp70 and Hsp90 were found in the CD9/TSG101/AchE-positive pellet but not in the CD9/TSG101/AchE-negative supernatant (Fig. 1d). Thus, tumor cell-released Hsp70 and Hsp90 were associated with CD9/TSG101/AchE-positive EVs. Next, we observed that the tumor cells that released high levels of Hsp70 and Hsp90 shown in Fig. 1a also released high levels of EVs quantified by AchE activity^21, 25^ (Fig. 1e, protein content of the EVs are shown in Supplementary Fig. 3B). Similarly, high levels of serum EVs were detected in LLC tumor-bearing and Apc^min/+^ mice that possessed high levels of serum Hsp70/90, but not in EL4 tumor-bearing mice whose serum Hsp70/90 levels were not significantly elevated (Fig. 1f). Remarkably, the extent of EV increase in cachectic tumor-bearing mice was similar to that of Hsp70/90 increase. Further, we treated the cell-conditioned media with the detergent Brij98 that solubilizes EVs^26^, which resulted in ~4–5-fold increases in ELISA-detected Hsp70 and Hsp90α as expected for vesicle-associated proteins (Supplementary Fig. 3C). Brij98 treatment of serum from tumor-bearing mice also robustly increased detection of Hsp70 and Hsp90 (Supplementary Fig. 3D) as compared to Fig. 1b. Thus, for accurate measurement of total circulating Hsp70/90 solubilization of EVs is necessary, and from this point on we included Brij98 treatment before ELISA measurement of tumor-released Hsp70/90 in all experiments. These data indicate that cachectic tumor cells release high levels of Hsp70 and Hsp90 that are associated with EVs.

### Elevated circulating Hsp70/90 induce muscle wasting

**Fig. 2:**
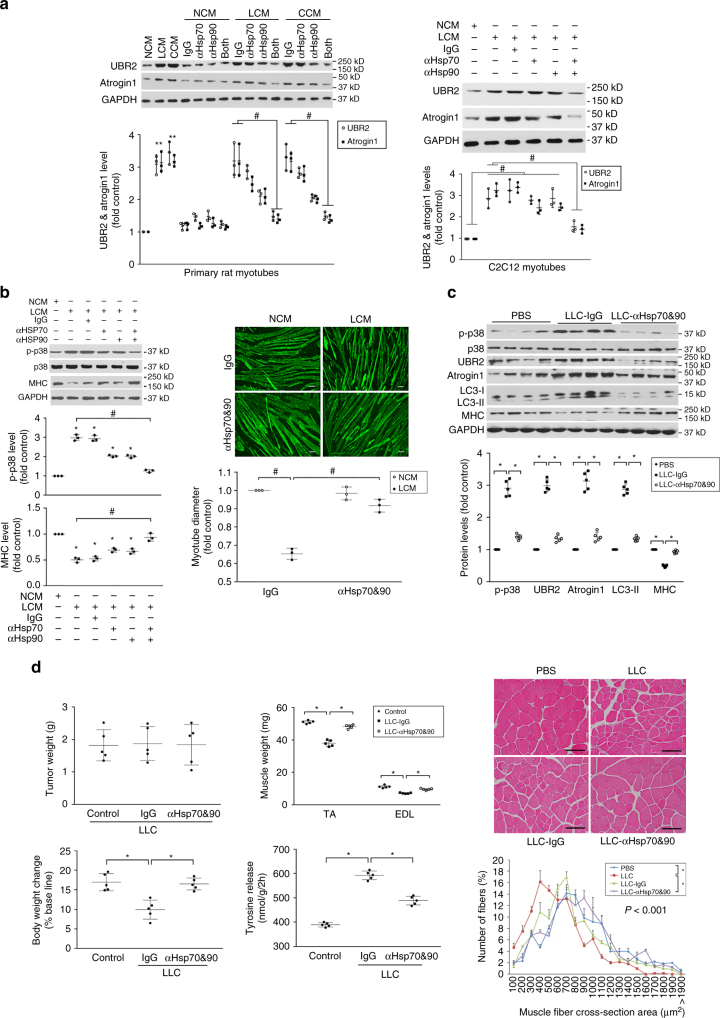
Elevated circulating Hsp70 and Hsp90 are critical to LLC tumor-induced catabolic response and muscle wasting. **a** Neutralizing antibodies of Hsp70 and Hsp90 block tumor cell-conditioned media-induced upregulation of atrogin1 and UBR2 in myotubes. Primary rat myotubes (*left*) or C2C12 myotubes (*right*) were treated for 8 h with NCM (NL20-conditioned medium), LCM (LLC-conditioned medium) or CCM (C26-conditioned medium) that were pre-incubated with pre-immune IgG (control) or neutralizing antibodies against Hsp70 and/or Hsp90 (100 ng/ml each) as indicated. Levels of atrogin1 and UBR2 in cell lysate were analyzed by western blotting. Data (*n* = 3) were analyzed by analysis of variance (ANOVA). * denotes a difference (*P* < 0.05) from NCM-treated control. ^#^ denotes a difference between bracketed columns. **b** Neutralizing antibodies of Hsp70 and Hsp90 block myotube atrophy induced by tumor cell-conditioned media. C2C12 myotubes were treated as described in **a** and analyzed for p38 MAPK activation (at 1 h) and MHC loss (at 72 h) by western blotting, and myotube diameter of MHC-stained myotubes (at 72 h). Data (*n* = 3) were analyzed by ANOVA. * denotes a difference (*P* < 0.05) from NCM-treated control. ^#^ denotes a difference tween bracketed columns. **c** Neutralizing antibodies of Hsp70 and Hsp90 prevent muscle catabolism in LLC tumor-bearing mice. Mice implanted with LLC cells were administered neutralizing antibodies against Hsp70 and Hsp90 (or pre-immune IgG as control) subcutaneously through osmotic pumps from day 7 at 3 μg/day each for 14 days and analyzed on day 21 for the activity of catabolic pathways in TA. Data (*n* = 5) were analyzed by ANOVA. * denotes a difference (*P* < 0.05). **d** Neutralizing antibodies of circulating Hsp70 and Hsp90 in LLC tumor-bearing mice prevent muscle wasting. Mice derived above in **c** were further analyzed for tumor volume and parameters of muscle wasting. Body weight baseline was day 0 of tumor cell implant. *Scale bar*, 100 μm. Data (*n* = 5) were analyzed by ANOVA or *χ* ^2^ analysis (for CSA). * denotes a difference (*P* < 0.05)

To determine whether elevated circulating Hsp70 and Hsp90 are responsible for the catabolic activity of tumor cells, we treated primary rat and C2C12 myotubes with conditioned medium of LLC (LCM) or C26 (CCM) cells with or without the inclusion of neutralizing antibody against Hsp70 and/or Hsp90, and monitored levels of E3 ligases atrogin1 and UBR2. We observed in both primary rat and C2C12 myotubes that the neutralizing antibodies partially blocked upregulation of atrogin1 and UBR2 when used alone, and abolished atrogin1 and UBR2 upregulation when used in combination (Fig. 2a). These data indicate that both Hsp70 and Hsp90 are required for the catabolic activity of LCM and CCM. Since C2C12 myotubes responded to the conditioned media in a similar manner as primary myotubes, we only used C2C12 myotubes for the other in vitro experiments in this study. Because LCM induces myotube catabolism through rapid activation of p38 MAPK, which activates C/EBPβ to upregulate *atrogin1* and *UBR2* ^4, 5, 10^, we examined whether LCM-induced p38 MAPK activation was dependent on Hsp70 and Hsp90. In an additive fashion, neutralizing antibodies of Hsp70- and Hsp90-blocked LCM-induced activation of p38 MAPK, and prevented myosin heavy chain (MHC) loss and myotube atrophy as measured by the diameters of myotubes (Fig. 2b). Thus, Hsp70 and Hsp90 are critical to LCM-induced p38 MAPK activation and myotube catabolism. To evaluate the in vivo role of elevated circulating Hsp70 and Hsp90 in cancer-induced muscle wasting, we administered neutralizing antibodies against Hsp70 and Hsp90 systemically to LLC tumor-bearing mice. Consistent with their effect in vitro, these neutralizing antibodies blocked tumor-induced catabolic response measured by activation of p38 MAPK, upregulation of atrogin1 and UBR2, increase of autophagy marker LC3-II and loss of MHC in tibialis anterior (TA, Fig. 2c). Consequently, without affecting tumor volume the neutralizing antibodies abrogated the development of muscle wasting as measured by body and muscle weight, muscle strength (grip strength), proteolysis (tyrosine release from extensor digitorum longus (EDL)) and muscle fiber cross-sectional area (CSA) (Fig. 2d).

**Fig. 3:**
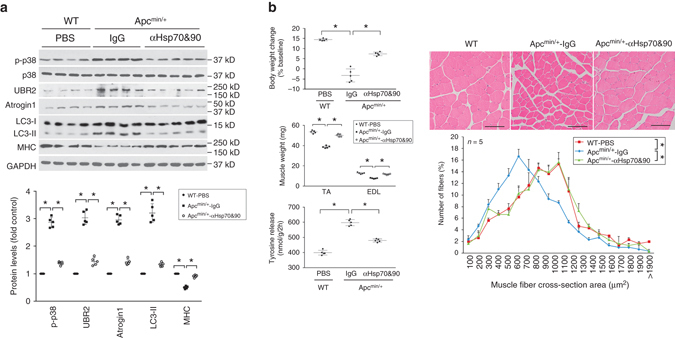
Elevated circulating Hsp70 and Hsp90 are critical to the development of muscle wasting in Apc^min/+^ mice. **a** Neutralizing antibodies of Hsp70 and Hsp90 block the activation of catabolic pathways in the muscle in Apc^min/+^ mice. Pre-cachectic Apc^min/+^ mice (16 weeks of age) were administered with neutralizing antibodies against Hsp70 and Hsp90 or pre-immune IgG subcutaneously via osmotic pumps at 3 μg/day each for 4 weeks. Wild-type mice in the same background (C57BL/6) were administered PBS for comparison. At 20 weeks of age the mice were killed and analyzed for activity of the catabolic pathways in TA. **b** Neutralizing antibodies of Hsp70 and Hsp90 attenuated muscle wasting in Apc^min/+^ mice. Mice derived from **a** were further evaluated for muscle wasting. Body weight baseline was at 12 weeks of age. *Scale bar*, 100 μm. Data (*n* = 5) were analyzed by ANOVA or *χ* ^2^ analysis (for CSA). * denotes a difference (*P* < 0.05)

To determine whether elevated circulating Hsp70 and Hsp90 are necessary for muscle wasting caused by diverse types of cancer, we evaluated whether muscle wasting in Apc^min/+^ mice is dependent on circulating Hsp70 and Hsp90. Administration of neutralizing antibodies of Hsp70 and Hsp90 to Apc^min/+^ mice from 16 to 20 weeks of age attenuated muscle catabolism (Fig. 3a) and development of muscle wasting (Fig. 3b), without altering tumor burden (Supplementary Fig. 4A). Together, the above data support a key role of elevated circulating Hsp70 and Hsp90 in the development of muscle wasting in vitro and in vivo caused by diverse cachexia-inducing tumors.

**Fig. 4:**
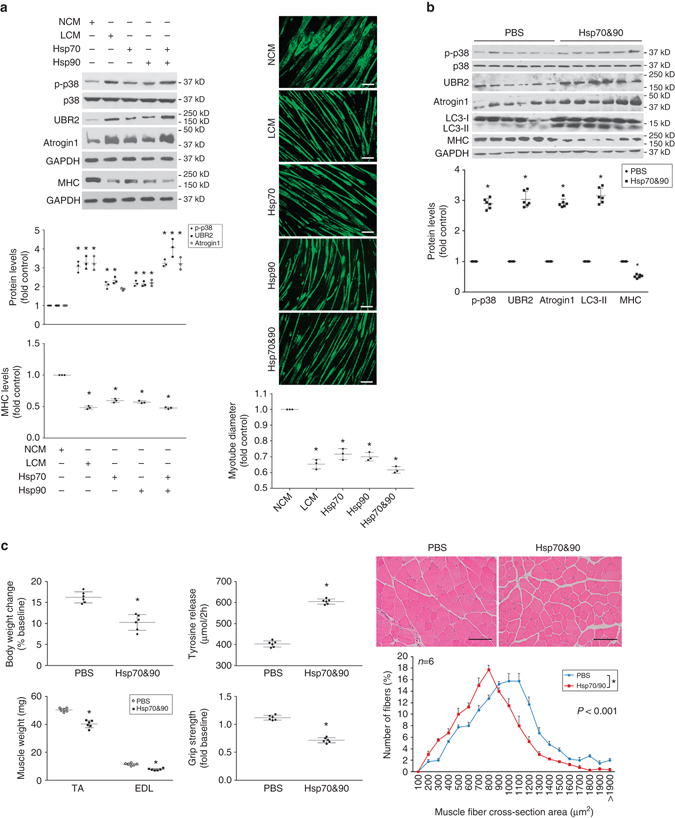
Administration of Hsp70 and Hsp90 causes muscle wasting by activating muscle catabolism. **a** Exogenous Hsp70 and Hsp90 induce a catabolic response in myotubes. C2C12 myotubes were treated with rHsp70 and/or rHsp90 (100 ng/ml each, controlled with PBS) as indicated, and LCM for comparison. Activation of catabolic pathways was analyzed as described above. Data (*n* = 3) were analyzed by analysis of variance. * denotes a difference from NCM-treated control (*P* < 0.05). **b** Systemically administered Hsp70 and Hsp90 activate muscle catabolism in mice. C57BL/6 mice were injected (i.p.) with rHsp70 and rHsp90 (each at 100 μg/kg/3 days, controlled with PBS) for five times. On day 15, activity of catabolic pathways in TA was analyzed. Data (*n* = 6) were analyzed by Student *t*-test. * denotes a difference from PBS-treated control (*P* < 0.05). **c** Systemically administered Hsp70 and Hsp90 cause muscle wasting in mice. Mice from **b** were further analyzed for muscle wasting. *Scale bar*, 100 μm. Data (*n* = 6) were analyzed by Student *t*-test or *χ* ^2^ analysis (for CSA). * denotes a difference from NCM- or PBS-treated control (*P* < 0.05)

Conversely, to evaluate whether elevated circulating Hsp70 and Hsp90 were sufficient for the development of muscle wasting, we treated myotubes with endotoxin-free recombinant Hsp70 (rHsp70) and/or Hsp90α (rHsp90), and found that rHsp70 and rHsp90 recapitulated the catabolic effect of LCM in an additive fashion (Fig. 4a). Notably, rHsp70 and rHsp90 activated p38 MAPK within 1 h, confirming that they induce muscle catabolism through their direct action on muscle cells without the need for new protein synthesis by muscle cells or input from immune cells. In addition, systemic administration of rHsp70 and rHsp90 to mice, which significantly increased serum Hsp70 and Hsp90 (Supplementary Fig. 5), induced muscle catabolism (Fig. 4b) and muscle wasting (Fig. 4c) similar to that observed in cachectic tumor-bearing mice. Thus, elevated circulating Hsp70 and Hsp90 are necessary and sufficient to induce muscle wasting in tumor hosts.

### Tumor cell-expressed Hsp70/90 are critical to muscle wasting

**Fig. 5:**
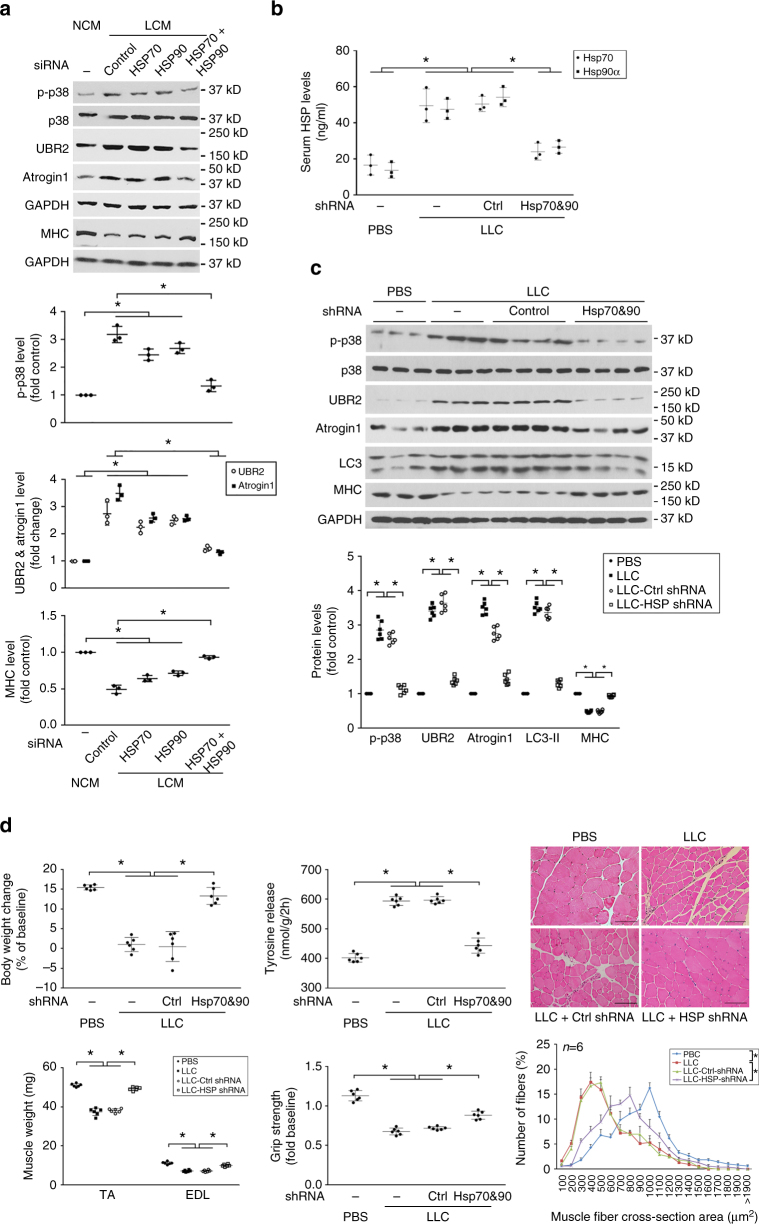
Tumor-induced muscle wasting depends on tumor cell-expressed Hsp70 and Hsp90. **a** Conditioned medium of Hsp70- and Hsp90-deficient LLC cells do not induce myotube catabolism. C2C12 myotubes were treated with LCM from LLC cells transfected with random or Hsp70/Hsp90-specific siRNA as indicated. Catabolic response was evaluated as described in Fig. 2. Data (*n* = 3) were analyzed by analysis of variance (ANOVA). * denotes a difference (*P* < 0.05). **b** Serum Hsp70 and Hsp90 levels in mice bearing Hsp70- and Hsp90-deficient LLC tumor remain unchanged. LLC cells were transduced with lentivirus encoding Hsp70 and Hsp90-specific shRNA, or lentivirus carrying empty vector as control. Mice were implanted with Hsp70&90-deficient or control LLC cells. On day 21 serum levels of Hsp70 and Hsp90 were analyzed after Brij98 treatment. Data (*n* = 6) were analyzed by ANOVA. * denotes a difference (*P* < 0.05). **c** Hsp70&90-deficient LLC tumor does not induce muscle catabolism in mice. TA of mice derived from **b** was analyzed by western blotting for markers of catabolic pathways. Data (*n* = 6) were analyzed by ANOVA. * denotes a difference (*P* < 0.05). **d** Hsp70&90-deficient LLC tumor do not cause muscle wasting in mice. Mice derived from **b** were further analyzed for muscle wasting. *Scale bar*, 100 μm. Data (*n* = 6) were analyzed by ANOVA or *χ* ^2^ analysis (for CSA). * denotes a difference (*P* < 0.05)

To determine whether the capacity of tumor to induce muscle wasting depends on its expression of Hsp70 and Hsp90 we knocked down *Hsp70* and/or *Hsp90* expression in LLC cells with siRNA (Supplementary Fig. 6A), which blocked high-level release of Hsp70 and Hsp90 by these cells (Supplementary Fig. 6B). Conditioned media of Hsp70- and/or Hsp90-deficient LLC cells lost the ability to induce the catabolic response in myotubes in an additive fashion (Fig. 5a). We also stably knocked down *Hsp70* and *Hsp90* in LLC cells by expressing specific shRNAs in LLC cells (Supplementary Fig. 6C). Mice bearing Hsp70/90-deficient LLC tumor did not manifest increased serum Hsp70/90 levels (Fig. 5b). Consequently, Hsp70/90-deficient LLC tumor did not induce muscle catabolism (Fig. 5c) and muscle wasting (Fig. 5d). Notably, Hsp70/90 deficiency did not significantly alter tumor burden (Supplementary Fig. 4B). These data indicate that tumor cell-expressed Hsp70 and Hsp90 are critical to the development of muscle wasting.

### Tumor-released Hsp70/90-expressing EVs cause muscle wasting

**Fig. 6:**
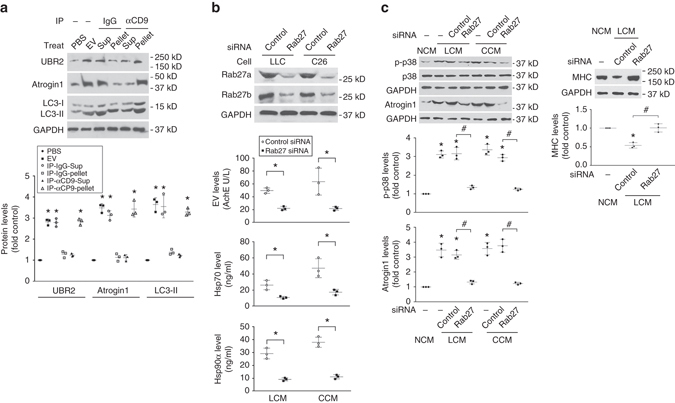
Tumor cell-released Hsp70/90-expressing EVs induce muscle catabolism in myotubes. **a** CD9-positive EVs induce catabolic response in C2C12 myotubes. EVs isolated from LCM were subjected to immunoprecipitation (IP) against CD9 with pre-immune IgG as control. Resulted pellet and supernatant (Sup) were used to treat C2C12 myotubes and compared with PBS and input EVs for catabolic activity using western blotting. Data (*n* = 3) were analyzed by analysis of variance (ANOVA) and * denotes a difference from PBS treatment (*P* < 0.05). **b** Hsp70/90 release by cachexia-inducing tumor cells is dependent on EV release. *Rab27a* and *Rab27b* were knocked down by transfecting LLC and C26 cells with control or specific siRNAs. EVs and Hsp70/90 released into conditioned media were quantified after Brij98 treatment. EVs were quantified by AchE. Data (*n* = 3) were analyzed by Student *t*-test. **c** Tumor cell-induced catabolic response in myotubes is dependent on EV release. C2C12 myotubes were treated with conditioned media of Rab27-deficient LLC or C26 cells and analyzed for p38 MAPK activity (at 1 h), atrogin1 (at 8 h) and MHC levels (72 h). Conditioned medium of non-tumorigenic NL20 cells (NCM) was used as control. Data (*n* = 3) were analyzed by ANOVA. * denotes a difference (*P* < 0.05) from NCM-treated control. ^#^ denotes a difference between bracketed columns

**Fig. 7:**
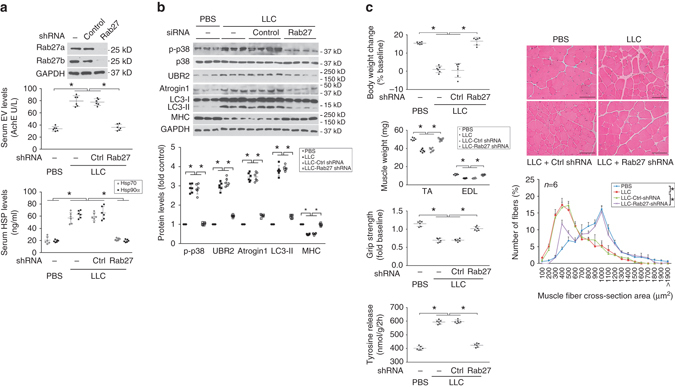
Tumor cell-released Hsp70/90-expressing EVs are critical to the development of muscle wasting in mice. **a** Elevation of serum Hsp70/90 in LLC tumor-bearing mice is dependent on EV release. LLC cells transduced with lentivirus encoding Rab27a- and Rab27b-specific shRNA or empty vector were analyzed for knockdown effect by western blotting (*top*). Mice were then implanted with Rab27-deficient or control LLC cells. In 21 days serum levels of EVs, Hsp70/90 were analyzed. **b** Rab27-deficient LLC tumor does not induce muscle catabolism in mice. Mice derived from **a** were analyzed for activity of the catabolic markers in TA. **c** Rab27-deficient LLC tumor does not induce muscle wasting in mice. Mice derived from **a** were analyzed for muscle wasting. *Scale bar*, 100 μm. Data (*n* = 6) were analyzed by analysis of variance or *χ* ^2^ analysis (for CSA). * denotes a difference (*P* < 0.05)

To determine whether tumor cell-released Hsp70/90-expressing EVs are critical to the development of muscle wasting, we treated C2C12 myotubes with EVs isolated from LCM. LLC-released EVs caused a catabolic response in a dose-dependent manner as indicated by upregulation of UBR2 and atrogin1 and increase in LC3-II (Supplementary Fig. 7). By treating C2C12 myotube with CD9-immunoprecipitated EVs it was further verified that CD9-positive EVs were responsible for the catabolic response induced by the EV preparation (Fig. 6a). Conversely, to disrupt tumor cell release of EVs we knocked down the expression of Rab27a and Rab27b, GTPases that control different steps of EV release^27^, in LLC and C26 cells utilizing siRNA. Loss of Rab27 abolished the release of EVs as well as Hsp70 and Hsp90 (Fig. 6b) without altering Hsp70/90 and AChE expression by tumor cells (Supplementary Fig. 8A), resulting in a blockade of the catabolic response in myotubes to tumor cell-conditioned media (Fig. 6c). Further, we observed that mice that were implanted with LLC cells in which Rab27a and Rab27b expression had been stably knocked down by shRNA prevented abnormal increase of serum EV as well as Hsp70/90 (Fig. 7a). We also verified that Hsp70/90 and AChE expression by the Rab27-deficient cells was not altered (Supplementary Fig. 8B). Without altered tumor burden (Supplementary Fig. 4C), mice bearing Rab27-deficient LLC tumor were spared from muscle catabolism (Fig. 7b) and muscle wasting (Fig. 7c). These data confirm that tumor cell-released Hsp70/90-expressing EVs are responsible for the development of muscle wasting by stimulating muscle catabolism. Thus, intervening tumor release of EVs could be an effective therapeutic strategy for cancer cachexia.

### Circulating Hsp70/90 induce muscle wasting through TLR4

**Fig. 8:**
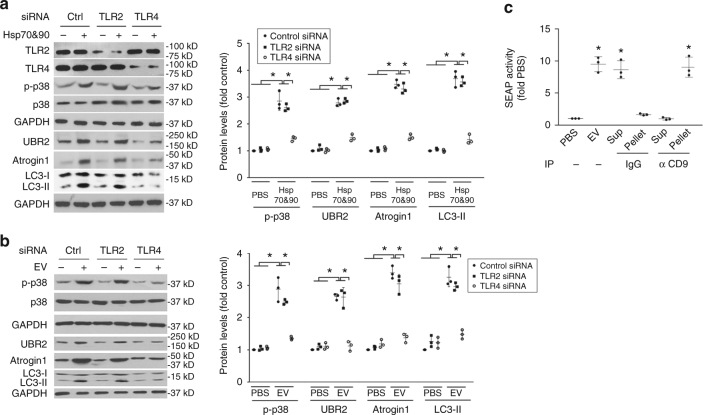
TLR4 mediates Hsp70/Hsp90-induced catabolic response in myotubes. **a** Hsp70 and Hsp90-induced catabolic response in myotubes is dependent on TLR4. C2C12 myoblasts were transfected with siRNA as indicated. After differentiation, myotubes were treated with rHsp70 and rHsp90 and analyzed for catabolic response as described above. **b** LLC-released EVs induce muscle catabolism in C2C12 myotubes through TLR4. C2C12 myotubes with either TLR2 or TLR4 knockdown were treated with EVs isolated from LCM (AchE 6 mU) and analyzed for catabolic response. **c** CD9-positive EVs activate TLR4 in reporter cells. EVs isolated from LCM were subjected to immunoprecipitation (IP) using an antibody against CD9 with pre-immune IgG as control. Resulting pellet and supernatant were used to treat TLR4 reporter cell line HEK-Blue hTLR4 for 24 h, and compared with original EVs. TLR4 activation was measured as SEAP activity. Data (*n* = 3) were analyzed by analysis of variance. * denotes a difference (*P* < 0.05)

**Fig. 9:**
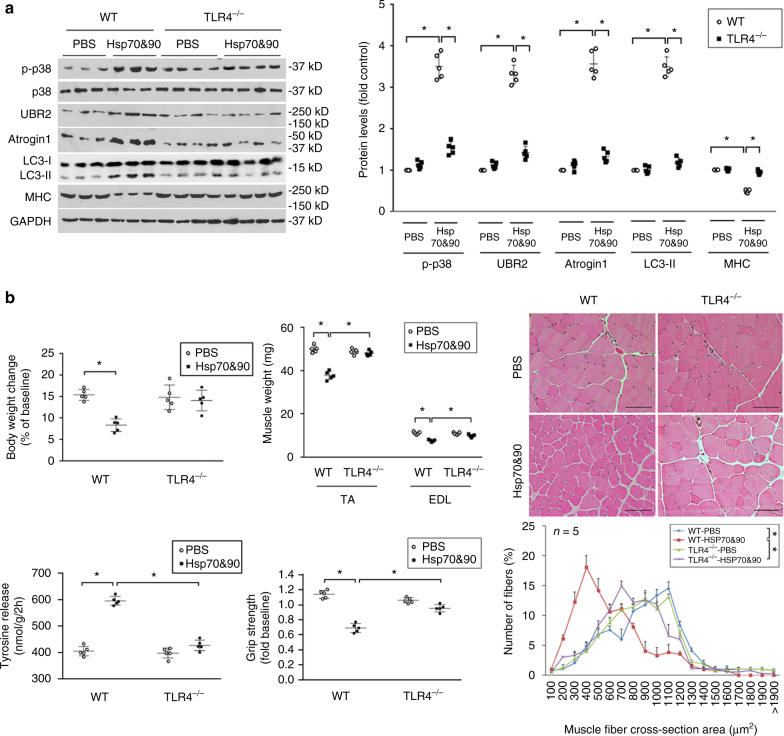
TLR4 is critical to Hsp70/Hsp90-induced muscle wasting in mice. **a** Hsp70 and Hsp90-induced muscle catabolism in mice is dependent on TLR4. Wild-type and TLR4 null mice were injected with rHsp70 and rHsp90 and analyzed for catabolic response in TA as described in Fig. 4. **b** Hsp70 and Hsp90-induced muscle wasting is TLR4-dependent. Wild-type and TLR4 null mice injected with rHsp70 and rHsp90 are further analyzed for muscle wasting. *Scale bar*, 100 μm. Data (*n* = 5) were analyzed by analysis of variance or *χ* ^2^ analysis (for CSA). * denotes a difference (*P* < 0.05)

To understand the mechanism through which extracellular Hsp70/90 mediates cancer-induced muscle wasting, we attempted to identify the plasma membrane receptor that mediates extracellular Hsp70/90-induced muscle catabolism. Extracellular Hsp70 and Hsp90 have been recognized as so called DAMPs^22^ and provoke innate immune response through activation of TLR2 and TLR4 on immune cells^28^. Particularly, EV-associated HSPs are capable of activating HSP receptors on recipient cells^18–21^. TLR2 and TLR4 are also expressed on skeletal muscle cells^29, 30^. Hence, we investigated whether Hsp70/90 induce muscle catabolism through the activation of TLR2/4 on myocytes by examining the effects of siRNA-mediated knockdown of *TLR2* or *TLR4* in C2C12 myotubes. We observed that the catabolic response of myotubes to rHsp70 and rHsp90 required TLR4 but not TLR2 (Fig. 8a), suggesting that Hsp70/90 induce muscle catabolism via activating TLR4 on muscle cells. To investigate whether Hsp70/90-expressing EVs induce muscle catabolism through TLR4, we treated myotubes with EVs isolated from LCM and observed an activation of catabolic response in a TLR4-dependent manner (Fig. 8b). To determine whether Hsp70/90-expressing EVs actually activate TLR4, we treated engineered HEK293 cells that overexpress TLR4 (HEK-Blue hTLR4) with LLC-released EVs and observed dose-dependent activation of TLR4-controlled reporter gene SEAP (Supplementary Fig. 9A). Utilizing CD9-immunoprecipitated EVs we verified that CD9-positive EVs were responsible for TLR4 activation (Fig. 8c). Further, rHsp70 and rHsp90 treatment of the reporter cells activated TLR4 (Supplementary Fig. 9B). Thus, Hsp70/90-expressing EVs mediate muscle catabolism by activating TLR4 on muscle cells. Next, by administering rHsp70 and rHsp90 to TLR4 null mice systemically, we confirmed that TLR4 was essential for Hsp70/90-induced muscle catabolism in vivo (Fig. 9a). Consequently, TLR4 deficiency rendered mice resistant to rHsp70 and rHsp90-induced muscle wasting (Fig. 9b). Thus, we conclude that tumor cell-released extracellular Hsp70 and Hsp90 induce muscle wasting by activating TLR4-mediated muscle catabolism.

### Tumor-released Hsp70/90 cause systemic inflammation

**Fig. 10:**
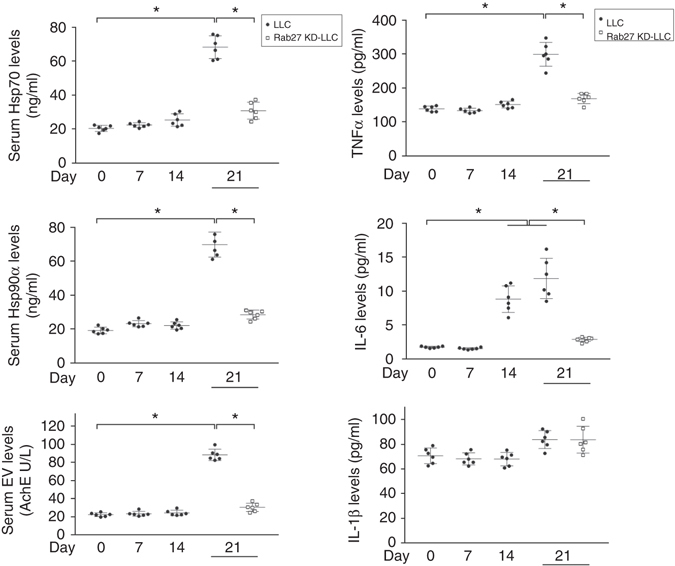
Tumor-released Hsp70 and Hsp90-expressing EVs mediate systemic increase of inflammatory cytokines. Sera from mice bearing LLC tumor with or without knockdown of Rab27 on days 0, 7, 14 and 21 of tumor cell implant were analyzed for levels of Hsp70, Hsp90 and EVs as described above, and TNFα, IL6 and IL-1β using Bio-Plex^®^ Multiplex Immunoassays. For Hsp70/90 measurement sera were treated with Brij98 before ELISA. Data (*n* = 6) were analyzed by analysis of variance. * denotes a difference (*P* < 0.05)

As an innate immune response, activation of TLR4 on myocytes and immune cells can increase their synthesis and secretion of inflammatory cytokines^31^ such as TNFα, IL-6 and IL-1β that promote muscle wasting, it is possible that tumor-released extracellular Hsp70 and Hsp90 cause the increase of circulating cytokines in tumor milieu. We thereby measured serum levels of TNFα, IL-6 and IL-1β in LLC tumor-bearing mice during the development of cachexia on days 0, 7, 14 and 21 of tumor implant as well as that of Hsp70/90 and EVs. As shown in Fig. 10, serum Hsp70/90 and EVs increased on day 21 when the mice developed cachexia. Serum TNFα also increased on day 21, while IL-6 increased from days 14 to 21, but IL-1β did not increase significantly during the entire period. Interestingly, elevation in serum TNFα and IL-6 were prevented in Rab27-deficient LLC tumor-bearing mice where serum Hsp70/90 and EV levels were not elevated, suggesting that elevated serum Hsp70 and Hsp90 mediate the systemic increase of inflammatory cytokines in tumor hosts. Thus, elevated serum Hsp70 and Hsp90 appear the source of tumor-induced system inflammation and should be considered key therapeutic targets of cancer cachexia.

Kir et al.^32^ reported previously that LLC tumor released high level of PTHrP, which induced muscle wasting through converting white adipose tissue to brown adipose tissue. We investigated whether PTHrP contributed to the muscle wasting in our cancer cachexia models. We found no abnormal release of PTHrP by LLC cells into culture medium as compared with non-tumorigenic cell lines (HPDE and NL20) and a tumorigenic cell line (EL4) that does not induce cachexia (Supplementary Fig. 10A). In addition, we found no increase of serum PTHrP in cachectic LLC tumor-bearing and Apc^min/+^ mice as compared to control mice (Supplementary Fig. 10B). Therefore, PTHrP does not appear to contribute to the muscle wasting in these models. There are multiple sub-strains of LLC cells with variable capacity in inducing cachexia. Given that the source of the LLC cells used by Kir et al. was unknown, the observed difference in abnormal PTHrP release suggests that their LLC was of a different sub-strain.

## Discussion

In this study, we demonstrate that constitutively releasing high levels of extracellular Hsp70 and Hsp90 through EVs is a common feature of diverse types of cachexia-inducing tumor cells, and that tumor cell-released extracellular Hsp70 and Hsp90 stimulate muscle catabolism resulting in muscle wasting. In addition, we show that extracellular Hsp70 and Hsp90 stimulate muscle catabolism through the activation of TLR4 on muscle cells. Further, we report that the increase of circulating inflammatory cytokines in tumor-bearing mice is dependent on tumor cell-released Hsp70/90. These findings depict tumor-released extracellular Hsp70 and Hsp90 as key cachexins responsible for muscle wasting in tumor-bearing mice.

EVs are cell-released membranous vesicles that can be divided into three subclasses: microvesicles, apoptotic blebs and exosomes. Exosomes (30–150 nm), the smallest of the three are released in large numbers by tumor cells into conditioned media and body fluids^33^. We detected tumor-released EVs from conditioned medium and serum that are consistent to exosomes in terms of size (~110 nm) and marker proteins (CD9/TSG101/AchE). We use the term ‘extracellular vesicles’ for these vesicles to conform to the recommendation by the International Society for Extracellular Vesicles^24^. Recently, exosomal adrenomedullin released by pancreatic cancer cells was shown to mediate pancreatic cancer-induced lipolysis, which may contribute to the fat tissue loss in cachexia^34^. The current study unveils a critical role of tumor cell-released EVs in mediating muscle wasting through the cargo proteins Hsp70 and Hsp90. Thus, tumor cell-released EVs appear to impact the metabolism of multiple organs in cachexia.

Utilizing multiple ‘loss and gain of function’ assays in vitro and in vivo, we demonstrated that tumor cell-released extracellular Hsp70 and Hsp90 are necessary and sufficient for the development of muscle wasting. Further, we showed that EV-associated extracellular Hsp70 and Hsp90 stimulate muscle catabolism by activating TLR4 on muscle cells. These data suggest that targeting circulating Hsp70/90, EV release or TLR4 may have translational values for the clinical intervention of cancer cachexia. In contrast to extracellular Hsp70/90 that are considered as DAMPs^22^, intracellular Hsp70/90 are cytoprotective against stressful stimuli as molecular chaperons. Therefore, although a number of pharmacological inhibitors of Hsp70/90 are available^35^, global inhibition of Hsp70/90 may not be desirable because intracellular Hsp70/90 in skeletal muscle may protect against diverse stimuli of muscle damage, dysfunction or atrophy^36–38^.

Intramuscularly, we demonstrated that Hsp70/90-expressing EVs stimulate muscle catabolism by activating TLR4 that mediates muscle wasting in diverse cancer cachexia models including LLC^39, 40^. We showed previously that TLR4 mediates muscle catabolism primarily through the activation of p38 MAPK, which in turn activates both the UPP and the ALP^39, 41^. We have also shown previously that the catabolic effect of p38 MAPK in muscle is exerted specifically by p38β MAPK through the activation of transcription factor C/EBPβ^4, 5^. The present study demonstrated that extracellular Hsp70 and Hsp90 activate TLR4 as well as p38 MAPK in muscle cells in an additive fashion leading to the activation of the UPP and the ALP, and resulting in loss of myofibrillar proteins, muscle mass and muscle strength. On the basis of these observations we propose that tumor-released extracellular Hsp70/90 induce muscle wasting primarily by activating the TLR4-p38β MAPK-C/EBPβ catabolic signaling pathway.

Systemic inflammation is one of the hallmarks of cancer cachexia, which was attributed to increased circulating inflammatory cytokines^2^. However, anti-cytokine strategies yielded mixed results in clinical trials for intervening cancer cachexia^11^. The present study shows that the increase of circulating cytokine TNFα and IL-6 in LLC tumor-bearing mice is dependent on tumor-released extracellular Hsp70/90, suggesting that TLR4 activation by extracellular Hsp70/90 increases synthesis and release of these cytokines as part of the host response to tumor^31^. Thus, elevation of extracellular Hsp70/90 appears the upstream driver of the systemic inflammation in tumor hosts, and inhibition of extracellular Hsp70/90-mediated signaling abrogates muscle catabolism through blocking their direct effect on muscle catabolism as well as indirect effect through cytokine release.

Elevated serum Hsp70 and Hsp90 have been reported in patients with cachexia-prone cancers including lung^42–47^, colorectal^48^ and pancreatic cancer^49^, but not in patients with breast cancer^50^ that is not highly prone to cachexia. Importantly, serum Hsp70 and Hsp90 levels in cancer patients increase with the development of pathological grade and clinical stage^43, 44, 46^, and the increase correlates with mortality in cancer patients^42, 48^, to which cachexia is a major determinant. Elevated EV levels have also been observed in the serum of lung, colorectal and pancreatic cancer patients^51–53^, which correlates with shorter survival^51^. However, these clinical studies did not examine whether elevation in serum Hsp70/90 and EVs correlated with cachexia. Because at least partially that non-standardized assays were used in these clinical studies, the reported values of basal serum Hsp70 in humans vary from 0.04^49^ to 8.5 ng/ml^47^, and basal serum Hsp90α levels vary from 17^50^ to 40 ng/ml^43^. The basal serum Hsp70 levels we measured in mice (~3 ng/ml, Fig. 1b) are similar to some of the clinical studies that reported 2–2.5 ng/ml^42, 46^. The basal serum Hsp90α levels we measured in mice (~4.5 ng/ml, Fig. 1b) were less than reported human values perhaps due to controlled strain and living conditions. Importantly, our findings of the 3–4-fold elevation of circulating Hsp70 and Hsp90α in cachectic tumor-bearing mice are similar to data from cancer patients^43, 46^. For the first time, our data link elevated circulating Hsp70/90 to cachexia and muscle wasting in mice. It is noteworthy that all previous clinical studies measured serum Hsp70/90 without solubilizing EVs. Thus, the actual serum content of the HSPs in cancer patients is likely much higher than reported as our data suggest (Supplementary Fig. 3D). The present study warrants further clinical studies to determine whether elevated serum Hsp70/90 levels are biomarkers of cachexia in cancer patients.

Tumor cell-released HSPs were previously shown to promote metastasis^19, 54^ and modulate immune cell activities^14, 21^. The current study uncovers a new role of circulating Hsp70 and Hsp90 as key cachexins that stimulate muscle catabolism. Nearly all body organs are negatively impacted by cancer, and cancer has been increasingly recognized as a systemic disease largely due to systemic inflammation. Our data suggest that extracellular Hsp70/90 are key inflammatory drivers in the tumor milieu. Thus, extracellular Hsp70/90 may be responsible for diverse pathologic effects of tumor on various organs either directly through activating their receptors, or indirectly through increasing circulating cytokines. Thus, our findings in the current study may have significance beyond cancer-induced muscle wasting and help understand how cancer affects whole body metabolism.

## Methods

### Muscle and tumor cell cultures

Murine C2C12 myoblasts (American Type Culture Collection (ATCC)) were cultured in growth medium (Dulbecco's Modified Eagle Medium (DMEM) supplemented with 10% fetal bovine serum) at 37 °C under 5% CO_2_. At 85–90% confluence, myoblast differentiation was induced by incubation for 96 h in differentiation medium (DMEM supplemented with 4% heat-inactivated horse serum) to form myotubes. Preconditioned medium from 48 h cultures of tumor cells including LLC, C26 adenocarcinoma cells (both from National Cancer Institute, Frederick, MD), H1299, BxPC-3, AGS and EL4 or non-tumorigenic cells NL20 and HPDE (all from ATCC) were collected (cell number counts are shown in Supplementary Fig. 1) and centrifuged (1000 × *g*, 5 min). The supernatant was used to treat myotube or HEK 293 cell cultures (25% final volume in fresh medium) when indicated, and replaced every 24 h. When indicated, neutralizing Hsp70 and Hsp90 antibodies (Enzo Life Technology, Farmingdale, NY) were included in tumor cell-conditioned medium with 30 min pre-incubation. All cell lines were verified to be mycoplasma free. Cell culture-based experiments were replicated independently for three times as estimated from previous data.

### Animal use

Experimental protocols were approved in advance by the institutional Animal Welfare Committee at the University of Texas Health Science Center at Houston. Mice were randomly grouped. For cachexia model generated by tumor cell xenograft, 100 μl LLC (National Institute of Cancer, Frederick, MD) or EL4 (ATCC) tumor cells (1 × 10^6^ cells), or an equal volume of vehicle (phosphate-buffered saline (PBS)) was injected subcutaneously into the right flanks of 7-week-old male C57BL/6 mice (Jackson Laboratory, Bar Harbor, Maine) or TLR4^−/−^ mice in C57BL/6 background^55^. Mice with tumor size smaller that 1 cm at the end of experiment were excluded. When indicated, neutralizing Hsp70 and Hsp90 antibodies (Enzo Life Technology) were administered from day 7 of LLC tumor implant, when the tumor became palpable, through a subcutaneously implanted Alzet® Osmotic Pump (Alzet, Cupertino, CA) containing 90 μl antibodies (45 μl each with a concentration of 1 mg/ml) or PBS as control. The pump released at a rate of 0.25 μl/h for 14 days (3 μg/day for each antibody). Development of cachexia was monitored by body weight change. Mice were euthanized after cachexia had been established on day 21 of LLC implant. Tumor was isolated and weighed (reported body weight was that excluded tumor weight). Recombinant Hsp70 and Hsp90 were injected (i.p.) to mice at 100 μg/kg per 3 days each for five times. Mice were euthanized on day 15 for analysis. TA and EDL muscles were then collected immediately for analyses. Male Apc^min/+^ mice (Jackson Laboratory, Bar Harbor, Maine) were administered with neutralizing Hsp70 and Hsp90 antibodies or PBS as control subcutaneously through an Alzet pump from 16 to 20 weeks of age, and euthanized at 20 weeks of age for analyses of muscle wasting. For pre-cachectic control Apc^min/+^ mice were euthanized and analyzed at 12 weeks of age. Mice that did not lose body weight at 16 weeks of age in comparison to 12 weeks of age were excluded. Polyp counting was conducted blindly. All mouse studies included five to six mice per group based on previous data.

### Proteomic analysis

LCM was concentrated 10 folds by 10k centrifugal filters (Millipore, Bedford, MA) and subjected to column chromatography (Mono Q ion exchange followed by gel filtration). Eluate fractions with catabolic activity determined as the ability to upregulate atrogin1 in C2C12 myotubes were separated by sodium dodecyl sulphate–polyacrylamide gel electrophoresis (SDS–PAGE) and subjected to silver staining (Sigma-Aldrich, St. Louis, MO). Proteins in the stained bands were identified with QStar Elite tandem LC MS/MS (The Applied Biosystems, Grand Island, NY) by the Proteomics Service Core at the University of Texas Health Science Center, Houston.

### Quantification of extracellular Hsp70 and Hsp90

Hsp70 and Hsp90 levels in various cell-conditioned media (concentrated 10 folds by centrifugation with 10k filters from Millipore) or mouse sera were analyzed by ELISA following the manufacturer’s instruction (Hsp70 EIA, Enzo Life Sciences; Hsp90α EIA, Cusabio Biotech, China). When indicated, Brij98 (0.5% v/v) was added to 100 µl of conditioned media or serum, and incubated for 30 min at 4 °C with gentle rotation to solubilize EVs before ELISA.

### EV isolation and quantitation

EVs in concentrated cell-conditioned media (described above) or sera were isolated using the ExoQuick^TM^ kit^56^ according to manufacturer’s protocol (System Biosciences, Mountain View, CA). Particle size of EV preparations was analyzed using ZetaView® Nanoparticle Tracking Analyzer (Particle Metrix GmbH, Meerbusch, Germany) according to the manufacturer’s manual. EVs were quantified by measuring the activity of AchE according to a published protocol^21^. Briefly, isolated EV preparations (20 µl) were suspended in 100 µl of PBS and incubated with 1.25 mM acetylthiocholine and 0.1 mM 5,5′-dithiobis (2-nitrobenzoic acid) in a final volume of 1 ml for 20 min at room temperature. Absorbance at 412 nm was monitored using the Synergy 2 Multi-Mode Microplate Reader (Biotek Instruments, Winooski, VT). Protein content in the EV preparations was measured by the Lowry assay (Bio-Rad, Hercules, CA).

### Electronic microscopy

EV preparations were resuspended in PBS and placed on a parafilm, which was then covered by a formvar carbon-coated nickel grid and allowed to sit for 60 min. The grids were washed with PBS, and the samples were fixed by 2% paraformaldehyde for 10 min. After wash with PBS, the EVs on the grid were stained with 2% osmium tetroxide for 1.5 h. The samples were post-fixed by 2.5% glutaraldehyde for 10 min, and contrasted by 2% uranyl acetate for 15 min. The samples were embedded using 0.13% methyl cellulose and 0.4% uranyl acetate for 10 min, and examined with a transmission electron microscope (JEM-1400, JEOL Ltd.) at 80 kV.

### Immunoprecipitation of EVs

Isolated EVs were suspended in PBS containing 1/100 phosphatase inhibitor cocktail (Sigma-Aldrich). The EV suspension (0.5 mg) was incubated with 2 μg of an antibody against CD9 (#SC-13118, Santa Cruz Biotechnology, Santa Cruz, CA) overnight at 4 °C, after pre-clearing with Pierce Protein A/G Agarose beads (Thermo Scientific). The mixture was then incubated with protein A/G Agarose beads for 2 h at 4 °C. The beads were centrifuged down and separated from supernatant. The pellet was washed five times with PBS. The supernatant and pellet were analyzed by western blot or used to treat cells. For cell treatment, the EVs were eluted from the pellet with 0.1 M glycine (pH 3.0) and dialyzed with ice-cold PBS overnight according to manufacturer’s protocol (Thermo Scientific).

### Transfection of siRNA

The siRNAs specific for TLR2 (CAU UAA GUC UCC GGA AUU A[dT][dT]), TLR4 (CCA UUG AUG AGU UCA GGU U[dT][dT]), Hsp70A1A (UGA ACC CCA CCA ACA CAG U[dT][dT] and Hsp70A1B GGU GGA GAU CAU CGC CAA C[dT][dT]), Hsp90α (ACC CAG ACC CAA GAC CAA C[dT][dT]), Hsp90β (GUG CAC CAU GGA GAG GAG G[dT][dT]), Rab27A (CGA UUG AGA UGC UCC UGG A[dT][dT]) and Rab27B (CUA AUU AAA GGG AGG AUA U[dT][dT]) were purchased from Sigma-Aldrich and control siRNA from Ambion (Austin, TX). Transfection of siRNA into C2C12 myoblasts or LLC cells was conducted using electroporation (5 μg/1 × 10^7^ cells) with the Nucleofector system (Lonza, Walkersville, MD) according to manufacturer’s protocol. In 24 h, myoblasts were transferred to differentiation medium, and LLC cells were transferred to fresh medium for further incubation to collect conditioned medium.

### shRNA expression in LLC cells

All shRNA constructs in pLKO.1 lentiviral vector were purchased from Sigma-Aldrich (MISSION®shRNA code: shHsp70, TRCN0000422257, TRCN0000347411; shHsp90A, TRCN0000321086; shHsp90B, TRCN0000071925; shRab27a, TRCN0000100575; shRab27b, TRCN0000100425) and packaged into viral particles according to manufacturer’s recommendation. Viruses were generated in TLA-293T cells from Thermo Fisher by co-transfecting four plasmids including the lentiviral vector, pMDLg/pRRE, pRSV-Rev and pMD2.G using Lipofectamine and Lipofectamine Plus reagent (2:3 ratio) from Invitrogen, Waltham, MA. At 48 and 72 h post transfection, virus-containing supernatants were collected for transduction in LLC cells. LLC cells transduced with shRNA were selected by puromycin.

### Recombinant Hsp70 and Hsp90

Recombinant human Hsp70 (derived from the baculovirus expression system) was purchased from Sigma-Aldrich (#SRP5190). Full-length human Hsp90α cDNA was subcloned into the pFastBacHT-C Baculovirus expression vector (Invitrogen), and expressed in Sf9 insect cells by the Vector Core at Baylor College of Medicine in Houston and purified with Ni-NTA agarose (Invitrogen).

### Fluorescence microscopy and histology study

C2C12 myotubes were stained with anti-MHC antibody (MF-20, Development Studies Hybridoma Bank at the University of Iowa, Iowa City, IA) followed by FITC-conjugated secondary antibody, and examined using a Zeiss Axioskop 40 microscope coupled to a Zeiss Axiocam MRM camera system that was controlled by Axiovision Release 4.6 imaging software. Acquired images were edited using the Photoshop software. To measure myotube diameter, fluorescein isothiocyanate (FITC)-MHC-stained myotubes were photographed under a fluorescence microscope at ×40. The diameters were measured in a total of 100 myotubes from ≥10 random fields using computerized image analysis (Scion Image, Frederick, MD, USA) as the average at three points along their length. The measurements were conducted in a blind fashion. Cross-sectional area of haemotoxylin and eosin (H&E) stained muscle sections was quantified using the ImageJ software (NIH).

### Polyp counts

Polyps in the intestine of Apc^min/+^ mice were counted as described by Mehl et al.^57^. Briefly, formalin-fixed intestinal sections from Apc^min/+^ mice were rinsed in PBS, stained in 0.1% methylene blue for 5 s, and counted by a single investigator who was blinded to the treatments. Polyps were counted under a dissecting microscope, using tweezers to pick through the intestinal villi and identify polyps. Polyps were categorized as >2, 1–2 and <1 mm.

### Western blot analysis

Proteins in muscle or cell lysate were separated by SDS–PAGE and transferred to nitrocellulose membranes. After incubation with a blocking buffer, the membranes were incubated with a primary antibody at 4 °C from 1 h to overnight. Horseradish peroxidase-conjugated secondary antibodies were used to locate the primary antibodies by the enhanced chemiluminescence method. Bands detected on the X-ray films were quantified using densitometry software (ImageQuant, GE). Antibodies to total (#9212L) and phosphorylated p38 MAPK (#4511S) were from Cell Signaling Technology (Danvers, MA). Hsp70 (#ADI-SPA-810-F) and Hsp90 (#ADI-SPA-830-F) antibodies were from Enzo Life Technology. Antibody for Atrogin1/MAFbx (#AP2041) was from ECM Biosciences (Versailles, KY). Antibody for MHC (#MAB4470) was from R&D Systems (Minneapolis, MN). Antibodies for UBR2 (#NBP1-45243), LC3 (#NB100-2220), Rab27A (#SC-74586), Rab27B (#NBP1-79631) were from Novus Biologicals (Littleton, CO). Antibodies for TLR2 (#SC-10739), TLR4 (#SC-10741), CD9 (#SC-13118), TSG101 (#SC-7964) and AchE (#SC-11409) were from Santa Cruz Biotechnology. Data were normalized to GAPDH (antibody #MAB374 was from Millipore). All primary antibodies were diluted by 1:1000 except for that for Rab27b by 1:2000. Supplementary Table 1 lists more information on the antibodies. Uncropped scans of blots shown in Results are included in Supplementary Fig. 11.

### Tyrosine release assay

Excised mouse EDL was pre-incubated for 30 min at 37 °C in Krebs Henseleit buffer saturated with 95% O_2_-5% CO_2_ (120 mM NaCl, 4.8 mM KCl, 25 mM NaHCO_3_, 2.5 mM CaCl_2_, 1.2 mM KH_2_PO_4_, and 1.2 mM MgSO_4_, pH 7.4, supplemented with 5 mM glucose, 5 mM HEPES, 0.1% bovine serum albumin, 0.17 mM leucine, 0.20 mM valine, 0.10 mM isoleucine, 0.1 U/ml insulin, and 0.5 mM cycloheximide; all from Sigma-Aldrich). EDL was then transferred into fresh buffer and incubated for 2 h. Tyrosine released into the buffer was determined by a fluorometric method^58^. Briefly, 0.5 ml of 30% trichloroacetic acid was added to 2 ml of buffer collected from tyrosine release assay, after 10 min incubation at room temperature, the mixture was centrifuged. Two milliliters of the deproteinized supernatant was then mixed with 1 ml each of nitrosonaphthol reagent and nitric acid reagent, and incubated at 55 °C for 30 min. After cooling to room temperature, 10 ml of ethylene dichloride was added and mixed vigorously to extract the unchanged nitrosonaphthol reagent. After centrifugation, the aqueous top layer was transferred to a cuvette and read in a TD-700 fluorometer (Turner Designs, Sunnyvale, CA, USA). Fluorescence of the tyrosine derivative resulting from its activation at 460 nm was measured at 570 nm. Concentration of tyrosine derivative was calculated using a standard curve. Rate of tyrosine release was normalized to the weight of the muscle as nanomoles of tyrosine per gram of muscle in 2 h.

### In vitro TLR4 activation assay

HEK-Blue™-hTLR4 cells (InvivoGen, San Diego, CA) are HEK293 cells co-transfected with the human TLR4, MD-2 and CD14 co-receptor genes, and an inducible SEAP (secreted embryonic alkaline phosphatase) reporter gene that is under the control of NF-κB and AP-1. HEK-Blue-hTLR4 cells and wild-type HEK 293 cells (as control) were treated with LCM derived EVs or Hsp70/90 for 24 h. SEAP activity in culture media was measured by using QUANTI-Blue as chromogenic substrate at 630 nm in Synergy 2 Multi-Mode Microplate Reader (Biotek Instruments) following manufacturer’s protocol.

### Bio-Plex^®^ multiplex immunoassays

Sera from LLC tumor-bearing mice were analyzed for specific cytokines utilizing Bio-Plex Pro™ Mouse Cytokine Th17 Panel A 6-Plex Group l (Bio-Rad Laboratories) according to manufacturer’s protocol. Cytokine concentrations were determined using the Bio-Plex 200 reader (software version 6.0, Bio-Rad Laboratories).

### Quantification of PTHrP

PTHrP level in tumor cell-conditioned medium or serum of tumor-bearing mice was determined by ELISA according manufacturer’s instruction (LifeSpan BioSciences, Seattle, WA).

### Statistical analysis

Data were presented as the mean ± S.D., and analyzed by the SigmaStat software (Systat Software, San Jose, CA) with Student *t*-test (data with two groups) or one-way analysis of variance (data with more than two groups) followed by a multiple comparison test chosen by the software. *χ* ^2^ analysis, using the FREQ procedure of SAS (SAS Institute Inc., Cary, NC), was used to compare muscle fiber cross-section area percentages among the various groups. A *P* value <0.05 was considered to be statistically significant.

### Study approval

Animal experimental protocols were approved in advance by the institutional Animal Welfare Committee at the University of Texas Health Science Center at Houston.

### Data availability

The authors declare that the main data supporting the findings of this study are available within the article and its Supplementary Information files. Extra data are available from the corresponding author upon request.

## Electronic supplementary material

Supplementary Information
