# Supplementary material for: Tumor induces muscle wasting in mice through releasing extracellular Hsp70 and Hsp90
Source: Nat Commun. 2017 Sep 19;8:589. doi: 10.1038/s41467-017-00726-x (PMC5605540; doi:10.1038/s41467-017-00726-x)
Supplement: Supplementary file 1 — Supplementary Information [file 41467_2017_726_MOESM1_ESM.pdf]

Description of Supplementary Files

File Name: Supplementary Information
Description: Supplementary Figures and Supplementary Table

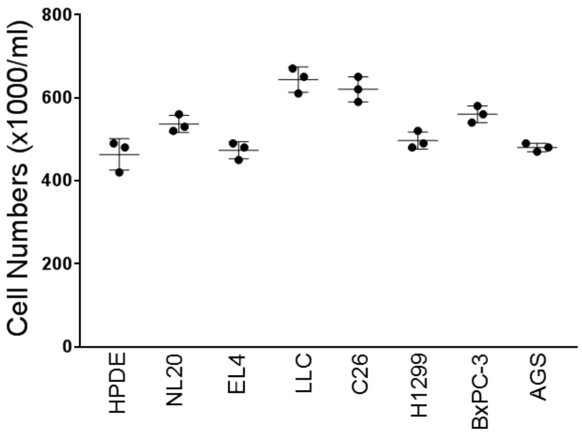

Supplementary Figure 1. Cell number at the time conditioned media were collected.

Conditioned media were collected from cultured cells at 48 h after seeding. Cell numbers were counted as shown.

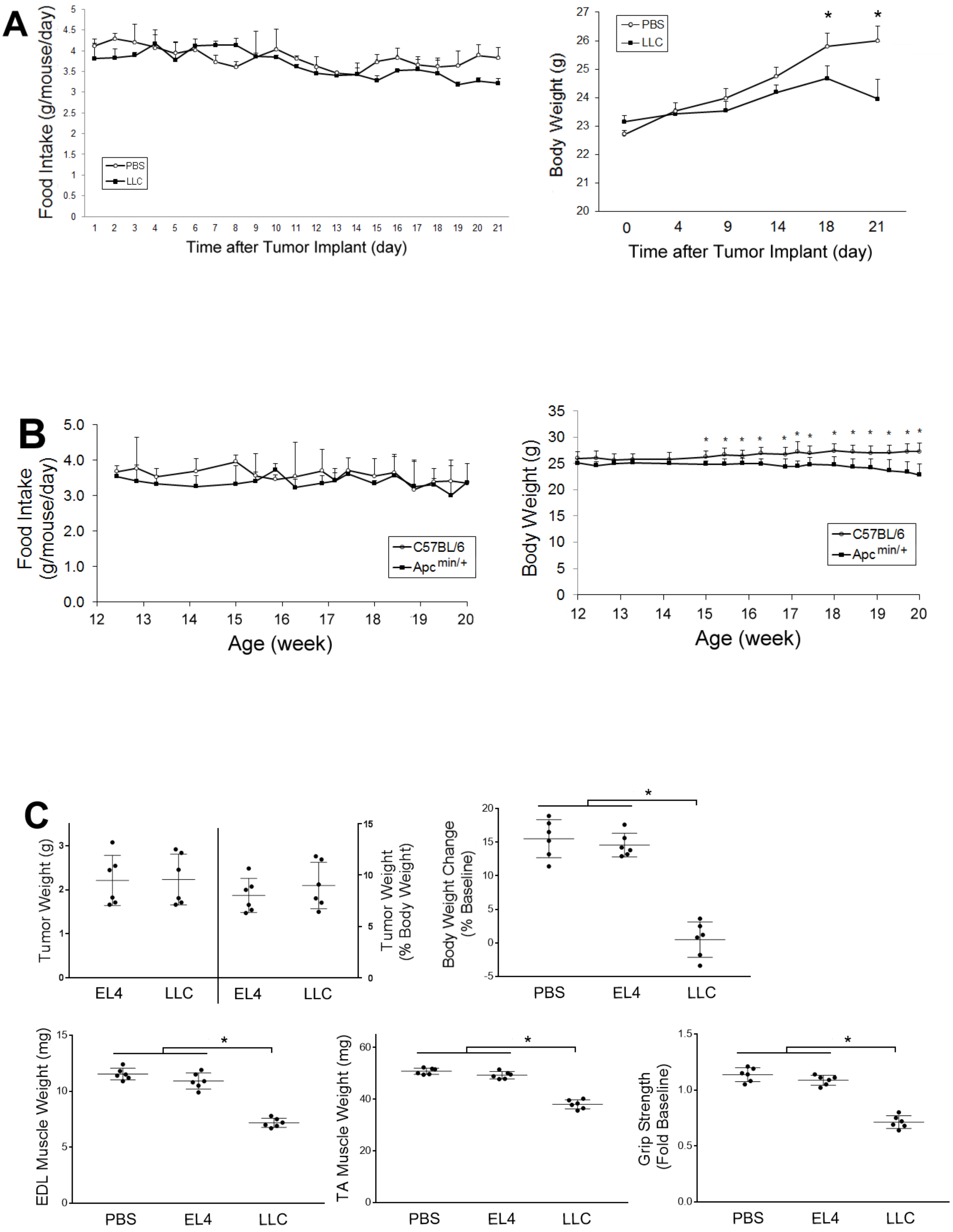
A
Food Intake (g/mouse/day)
PBS
LLC
Time after Tumor Implant (day)
Body Weight (g)
PBS
LLC
Time after Tumor Implant (day)
B
Food Intake (g/mouse/day)
C57BL/6
Apc min/+
Age (week)
Body Weight (g)
C57BL/6
Apc min/+
Age (week)
C
Tumor Weight (g)
Tumor Weight (% Body Weight)
EL4
LLC
EL4
LLC
Body Weight Change (% Baseline)
PBS
EL4
LLC
EDL Muscle Weight (mg)
PBS
EL4
LLC
TA Muscle Weight (mg)
PBS
EL4
LLC
Grip Strength (Fold Baseline)
PBS
EL4
LLC

Supplementary Figure 2. LLC-tumour-bearing and $Apc^{min/+}$ mice, but not EL4 tumour-bearing mice, develop cachexia and muscle wasting. (A) Development of cachexia in LLC tumour-bearing mice. LLC cells were implanted to C57BL/6 mice (7 weeks of age) subcutaneously (1×10^6 cells) or PBS as control. Food intake and body weight were monitored for 3 weeks. Data was analyzed by Student t test. **(B)** Development of cachexia in $Apc^{min/+}$ mice. Food intake and body weight were monitored in $Apc^{min/+}$ and wildtype mice from 12 weeks to 20 weeks of age. Data was analyzed by Student t test. **(C)** EL4 tumour-bearing mice do not develop cachexia and muscle wasting. EL4 cells or LLC cells were implanted to male C57BL/6 mice (7 weeks of age) subcutaneously (1×10^6 cells). Control mice were injected with PBS. In 21 days tumour weight (absolute and relative), body and muscle weight, as well as muscle strength were measured. Data were analyzed by ANOVA. * denotes a difference ($p < 0.05$).

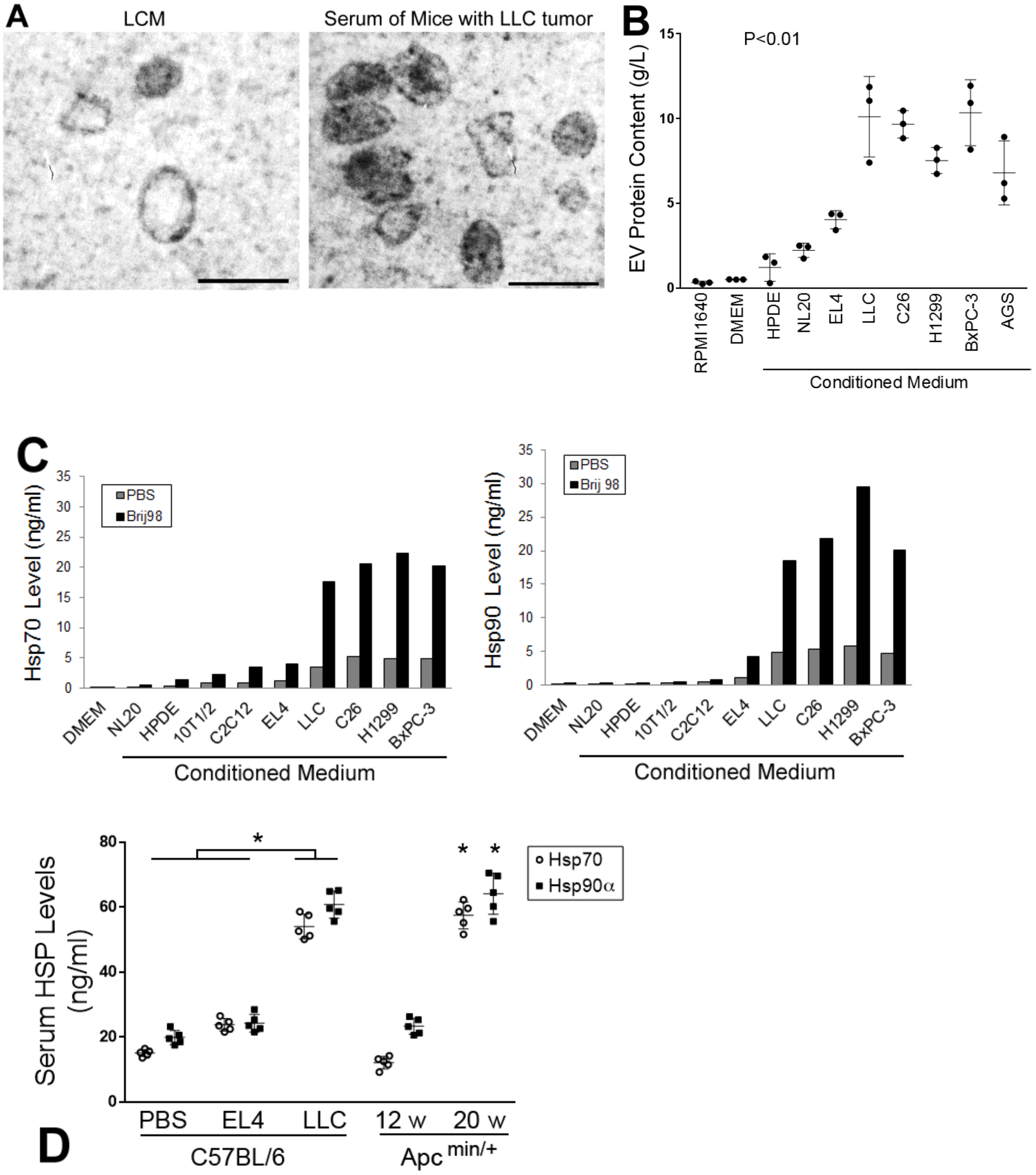

A
LCM
Serum of Mice with LLC tumor
B
P<0.01
EV Protein Content (g/L)
RPMI1640
DMEM
HPDE
NL20
EL4
LLC
C26
H1299
BxPC-3
AGS
Conditioned Medium
C
Hsp70 Level (ng/ml)
PBS
Brij98
DMEM
NL20
HPDE
10T1/2
C2C12
EL4
LLC
C26
H1299
BxPC-3
Conditioned Medium
Hsp90 Level (ng/ml)
PBS
Brij 98
Conditioned Medium
D
Serum HSP Levels (ng/ml)
Hsp70
Hsp90α
PBS
EL4
LLC
12 w
20 w
C57BL/6
Apc min/+

Supplementary Figure 3. Additional data on tumour cell-released EVs. (A) EVs isolated from LCM and the serum of cachectic LLC tumour-bearing mice (day 21 of tumour cell implant) were visualized by electron microscopy. Bar = 200 nm. **(B)** Protein content of tumour cell-released EVs shown in Figure 2E. **(C)** Solubilization of EVs increased detection of Hsp70 and Hsp90 in tumour cell-conditioned media. Cell conditioned media were subjected to incubation with detergent Brij98 (0.5% v/v) for 30 min at 4ºC to solubilize EVs with PBS as control. Hsp70 and Hsp90 levels in the media were then determination by ELISA. (D) Solubilization of EVs increased detection of Hsp70 and Hsp90 in serum of LLC tumor-bearing mice. Sera derived from Figure 1B were subjected to incubation with detergent Brij98 as described above prior to ELISA determination of Hsp70 and Hsp90.

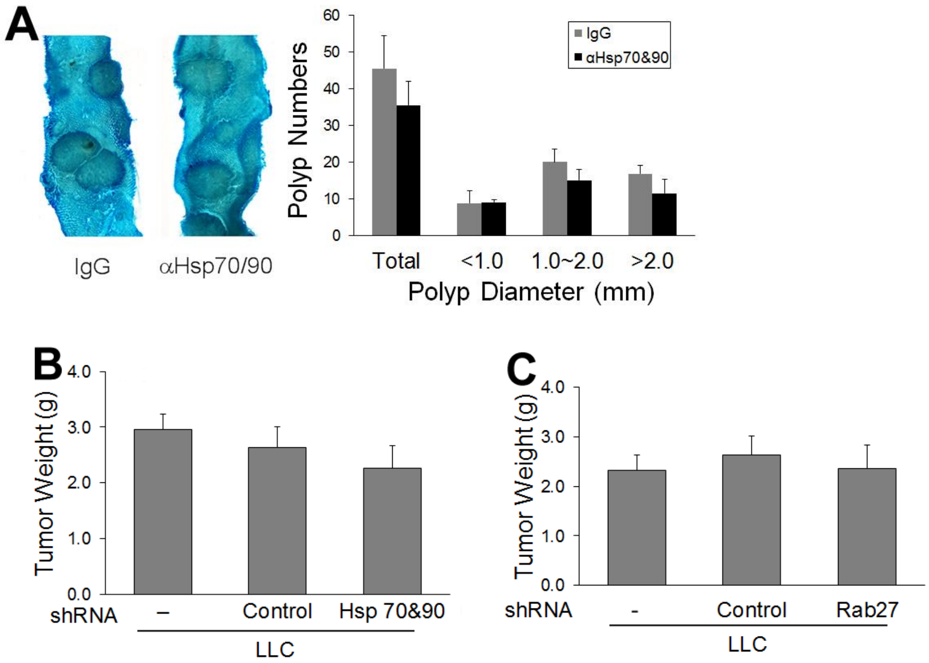

Supplementary Figure 4. Tumour burden in mice that received described interventions. **(A)** Tumour burden (polyp number) was not altered in $Apc^{min/+}$ mice that had been treated with antibodies against Hsp70 and Hsp90 as measured at the end of experiment (20 weeks of age). The left penal shows examples of stained intestine with polyps. The right panel shows the number of polyps. **(B)** Tumour burden (weight) was not altered in mice bearing LLC tumour in which Hsp70 and Hsp90α/β had been knocked down by shRNA as measured at the end of experiment (3 weeks after LLC cell implant). **(C)** Tumour burden (weight) was not altered in mice bearing LLC tumour in which Rab27a and Rab27b had been knocked down by shRNA as measured at the end of experiment (3 weeks after LLC cell implant). Data were analyzed by Student t test (A) or ANOVA (B and C), no difference was found ($p > 0.05$).

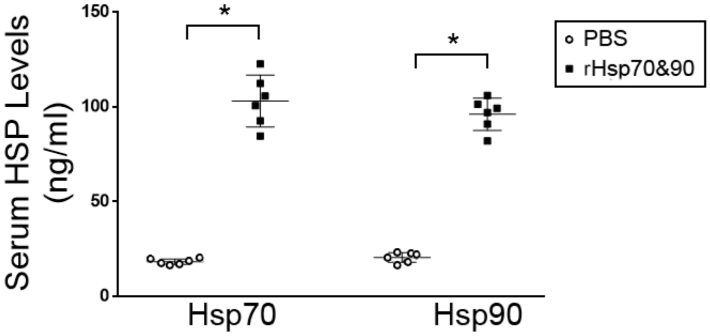

Supplementary Figure 5. Serum HSP levels increase in mice administered rHsp70 and rHsp90. Mice were injected with rHsp70 and rHsp90 (i.p.) at 100 μg/Kg/3 days each for 5 times with PBS as control. Serum Hsp70/90 levels were determined on day 15 by ELISA with Brij98 treatment. Data was analyzed by Student t test and * denotes a difference from PBC treatment ($p < 0.05$).

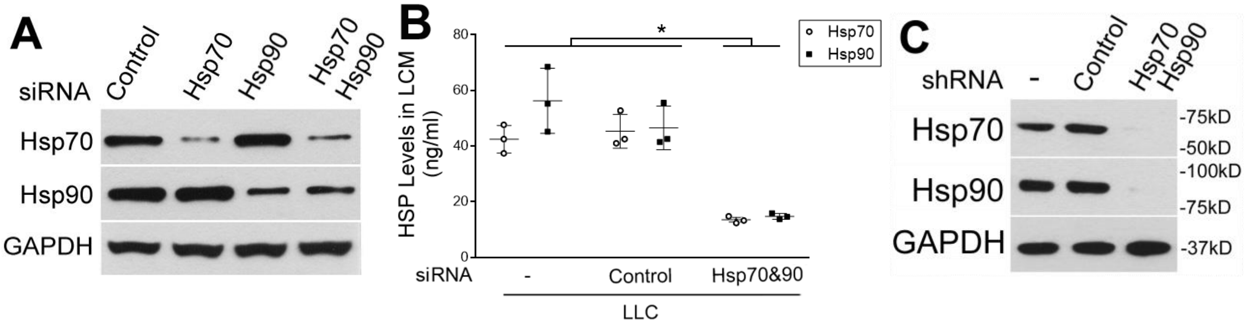

Supplementary Figure 6. Knockdown of *Hsp70* and in LLC cells by siRNA or shRNA. (A) LLC cells were transfected with control, Hsp70- and/or Hsp90α/β-specific siRNA. In 48 h, Hsp70 and Hsp90 knockdown were examined by Western blotting. **(B)** Effect of siRNA-mediated knockdown of Hsp70 and Hsp90 on their release into LLC cell-conditioned medium was determined by ELISA with Brij98 treatment. Data was analyzed by ANOVA. * denotes a difference ($p < 0.05$). **(C)** LLC cells were transduced with lentivirus encoding control or Hsp70- and Hsp90α/β-specific shRNA. In 72 h, Hsp70 and Hsp90 knockdown were examined by Western blotting.

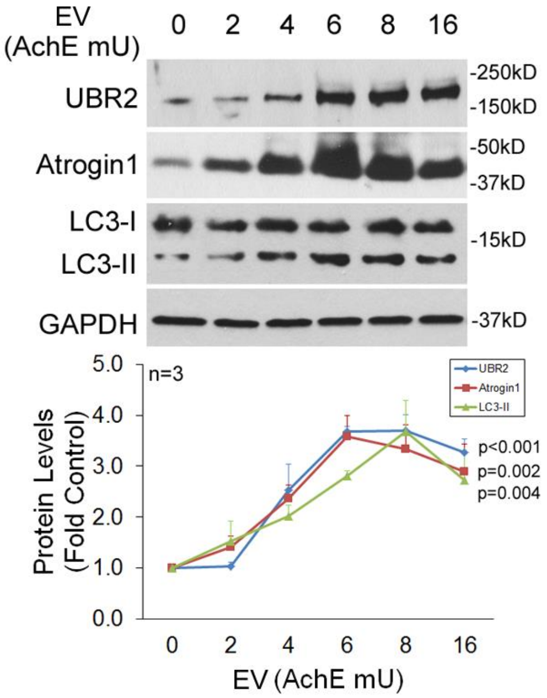

Supplementary Figure 7. LLC-released EVs induce myotube catabolism. C2C12 myotubes were treated with indicated quantities of EVs isolated from LCM for 8 h. Markers of muscle catabolism were analyzed in 8 h by Western Blotting. Data were analyzed by ANOVA.

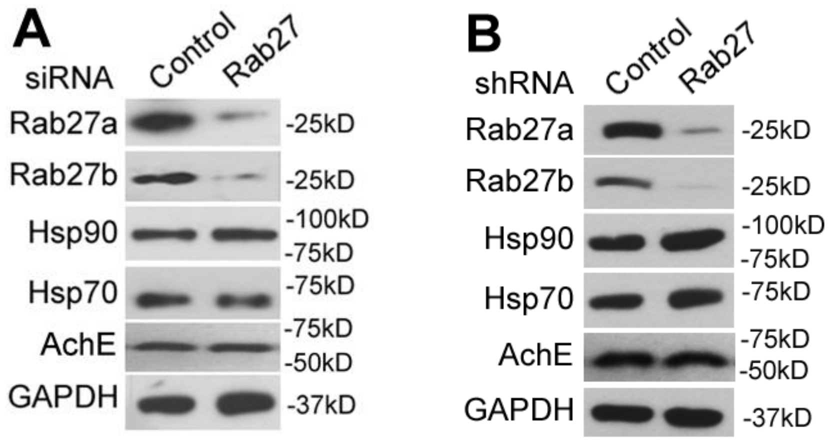

Supplementary Figure 8. Rab27 knockdown in tumour cells does not affect Hsp70, Hsp90 and AChE expression. LLC cells were transfected with *Rab27a* and *Rab27b*-specific siRNA **(A)** or transduced with lentivirus encoding *Rab27a* and *Rab27b*-specific shRNA **(B)** as described in Figure 6 and 7. Hsp70, Hsp90 and AChE levels in cell lysate were measured by Western blotting.

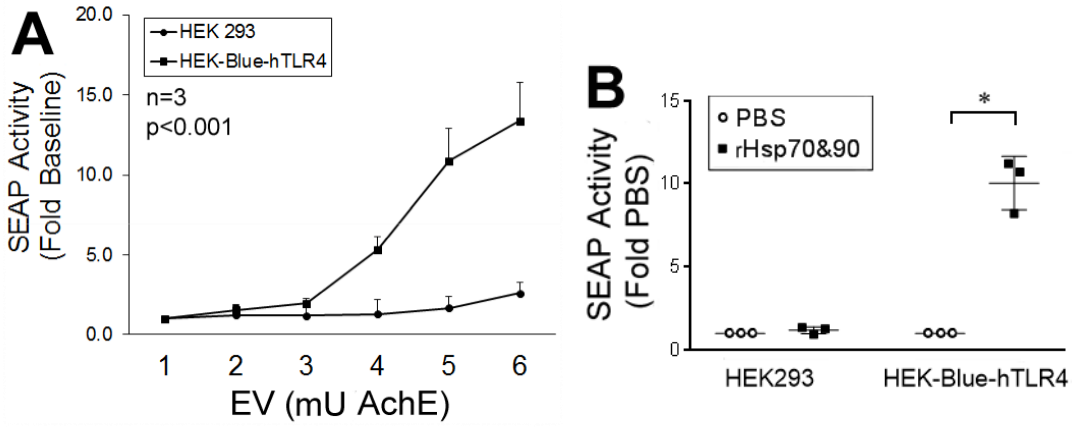

Supplementary Figure 9. LLC-released EVs and recombinant Hsp70/90 activate TLR4 in reporter cells. **(A)** LLC-released EVs activate TLR4 on reporter cells. TLR4 reporter cell line HEK-Blue hTLR4 (InvivoGen) was treated with EVs isolated from LCM with indicated quantities for 24 h and TLR4 activation was measured as the enzymatic activity of secreted embryonic alkaline phosphatase (SEAP). Data was analyzed by ANOVA. **(B)** HEK-Blue hTLR4 cells were treated with rHsp70 and rHsp90 (100 ng/ml each) for 24 h. TLR4 activation was measured as SEAP activity. Data was analyzed by Student t test. * denotes a difference ($p < 0.05$).

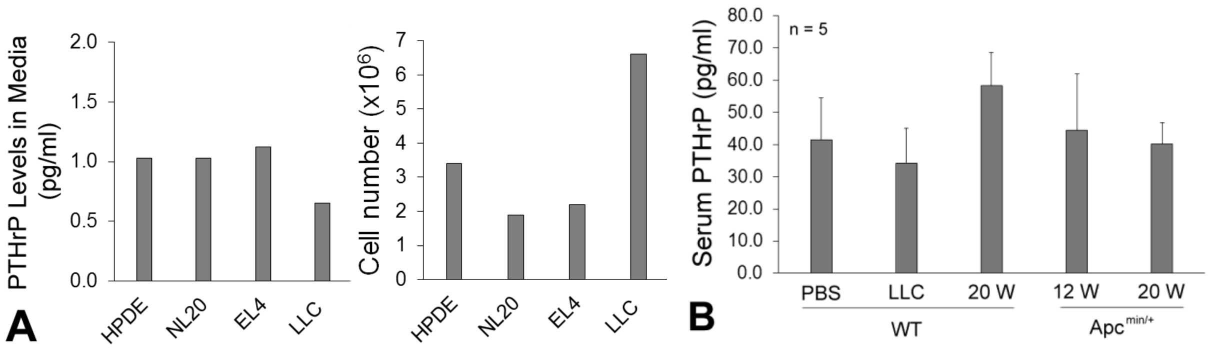

Supplementary Figure 10. PTHrP levels are not elevated in LCM and serum of cachectic tumor-bearing mice. (A) PTHrP levels are not elevated in LCM. Conditioned media of LLC and control cells collected at 48 h were analyzed by ELISA for PTHrP levels (left panel). Cell number at the time of collection is shown in the right panel. **(B)** PTHrP levels are not elevated in the serum of cachectic LLC tumor-bearing and $Apc^{min/+}$ mice. Serum was collected from LLC tumor-bearing mice and PBS-injected control C57BL/6 mice (wild type) on day 21 of tumor implant, as well as from $Apc^{min/+}$ mice at 12 and 20 weeks of age and control C57BL/6 mice at 20 weeks of age. PTHrP levels were determined by ELISA.

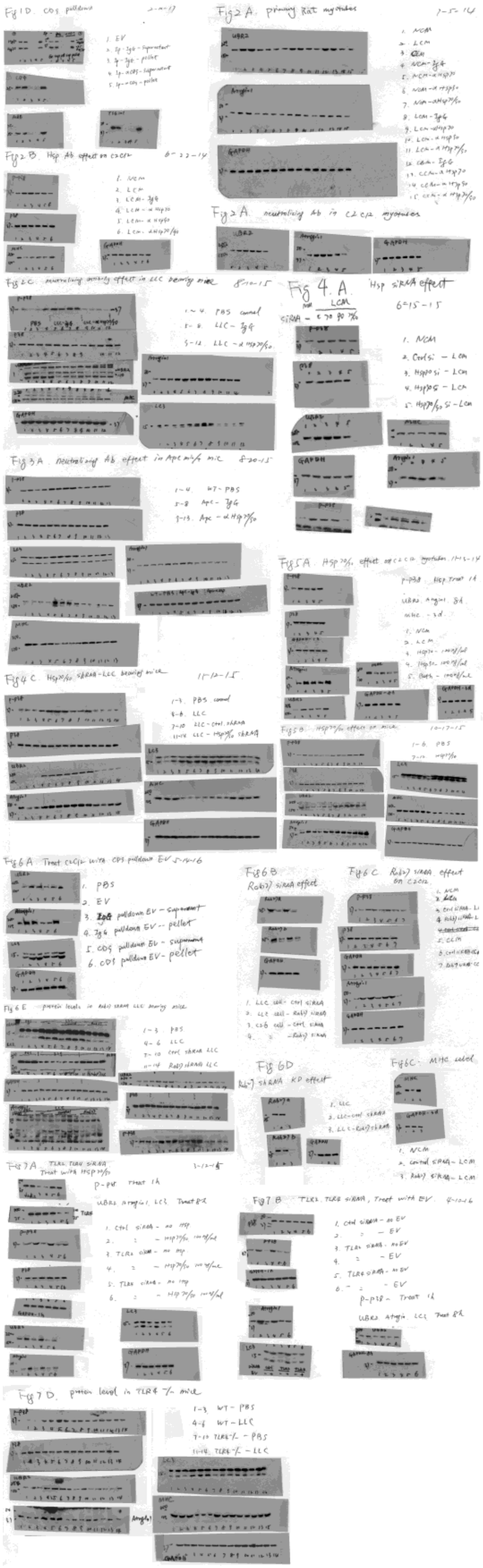

Supplementary Figure 11. Uncropped scans of Western blots.

Supplementary Table 1. Antibody profile.

Antibody	Species	Catalog Number	Provider	Validation Profile or Citation
p38	Rabbit	9212L	Cell Signaling Technology	Zhang, G. et al. *EMBO J*. 30, 4323-4335, 2011.
p-p38	Rabbit	4511S	Cell Signaling Technology	Zhang, G. et al. *EMBO J*. 30, 4323-4335, 2011.
Hsp70	Mouse	ADI-SPA-810-F	Enzo Life Sciences	https://www.antibodypedia.com/gene/27819/HSPA1A/antibody/2062317/ADI-SPA-810-F
Hsp90	Mouse	ADI-SPA-830-F	Enzo Life Sciences	https://www.antibodypedia.com/gene/3676/HSP90AA1/antibody/643848/ADI-SPA-830-F
Atrogin1	Rabbit	AP2041	ECM Biosciences	Puppa, M.J. et al. *FASEB J*. 28(2):998, 2014
MHC	Mouse	MAB4470	R&D Systems	https://www.antibodypedia.com/gene/32261/MYH14/antibody/2364757/MAB4470
UBR2	Goat	NBP1-45243	Novus Biologicals	https://www.antibodypedia.com/gene/30162/UBR2/antibody/1129672/NBP1-45243
LC3	Rabbit	NB100-2220	Novus Biologicals	https://www.antibodypedia.com/gene/30678/MAP1LC3B/antibody/70809/NB100-2220
Rab27b	Rabbit	NBP1-79631	Novus Biologicals	https://www.antibodypedia.com/gene/4487/RAB27B/antibody/583797/NBP1-79631
Rab27a	Rabbit	sc-74586	Santa Cruz Biotechnology	Poeter, M. et al. *Nat Commun*. 5: 3738, 2014.
TLR2	Rabbit	SC-10739	Santa Cruz Biotechnology	Lee, H.M., et al. *J. Clin. Immunol*. 29: 46-56, 2009.
TLR4	Rabbit	SC-10741	Santa Cruz Biotechnology	Pal, D., et al. *Nat. Med*. 18: 1279-1285, 2012.
CD9	Mouse	SC-13118	Santa Cruz Biotechnology	Gallart-Palau, X. et al. Sci. Rep. 5: 14664, 2015.
TSG101	Mouse	SC-7964	Santa Cruz Biotechnology	Song, X., et al. *J. Biol. Chem*. 291: 8453-8464, 2016.
AchE	Rabbit	SC-11409	Santa Cruz Biotechnology	Zhang, X., et al. *Autophagy* 10: 588-602, 2014.
GAPDH	Mouse	MAB374	EMD Millipore	https://www.antibodypedia.com/gene/3923/GAPDH/antibody/554563/MAB374
